# Association of Histological and Clinical Chorioamnionitis With Neonatal Sepsis Among Preterm Infants: A Systematic Review, Meta-Analysis, and Meta-Regression

**DOI:** 10.3389/fimmu.2020.00972

**Published:** 2020-06-05

**Authors:** Eduardo Villamor-Martinez, George A. Lubach, Owais Mohammed Rahim, Pieter Degraeuwe, Luc J. Zimmermann, Boris W. Kramer, Eduardo Villamor

**Affiliations:** Department of Pediatrics, Maastricht University Medical Center (MUMC+), School for Oncology and Developmental Biology (GROW), Maastricht, Netherlands

**Keywords:** chorioamnionitis, neonatal sepsis, immunomodulation, very preterm birth, extremely preterm birth, meta-analysis, meta-regression, systematic review

## Abstract

Chorioamnionitis (CA) is considered a key risk factor for very preterm birth and for developing early onset sepsis (EOS) in preterm infants, but recent data suggest that CA might be protective against late onset sepsis (LOS). We performed a systematic review and meta-analysis of studies exploring the association between CA and sepsis. A comprehensive literature search was performed in PubMed/MEDLINE and EMBASE, from their inception to December 1, 2018. A random-effects model was used to calculate odds ratios (OR) and 95% confidence intervals (CI). Sources of heterogeneity were analyzed by subgroup and meta-regression analyses. The following categories of sepsis were analyzed: EOS, LOS, unspecified onset sepsis (UOS), culture-proven, and clinical sepsis. CA was subdivided into clinical and histological chorioamnionitis. Funisitis was also analyzed. We found 3,768 potentially relevant studies, of which 107 met the inclusion criteria (387,321 infants; 44,414 cases of CA). Meta-analysis showed an association between any CA and any EOS (OR 4.29, CI 3.63–5.06), any LOS (OR 1.29, CI 1.11–1.54), and any UOS (OR 1.59, CI 1.11–1.54). Subgroup analysis showed that CA was associated with culture-proven EOS (OR 4.69, CI 3.91–5.56), clinical EOS (OR 3.58, CI 1.90–6.76), and culture-proven LOS (OR 1.31, CI 1.12–1.53), but not with clinical LOS (OR 1.52, CI 0.78–2.96). The presence of funisitis did not increase the risk of either EOS or LOS when compared with CA without funisitis. CA-exposed infants had lower gestational age (−1.11 weeks, CI −1.37 to −0.84) than the infants not exposed to CA. Meta-regression analysis showed that the lower gestational age of the CA group correlated with the association between CA and LOS but not with the association between CA and EOS. In conclusion, our data suggest that the positive association between chorioamnionitis and LOS may be modulated by the effect of chorioamnionitis on gestational age.

## Introduction

Very preterm birth is defined by a gestational age (GA) below 32 weeks, and extremely preterm birth is defined by a GA below 28 weeks (1). The etiological background of very/extremely preterm birth can be divided into two main categories: intrauterine infection/inflammation and placental vascular dysfunction (2–4). The first category is associated with chorioamnionitis (CA), preterm labor, premature rupture of membranes (PROM), placental abruption, and cervical insufficiency, whereas the second category is associated with gestational hypertensive disorders and condition known as fetal indication/fetal growth restriction (2–4). Belonging to the first group, CA is the maternal response to an intrauterine infection/inflammation and implies the presence of inflammatory cells in the extraplacental membranes (chorion and amnion) (5, 6). Acute CA generally represents the presence of intraamniotic infection or “amniotic fluid infection syndrome” but can also occur in the in the absence of proven infection (6). This may partly be due to lack of detection of some bacterial species by the culturing methods routinely employed (7, 8).

CA is not only considered to be a leading cause of very/extremely preterm birth but also a main factor in the development of subsequent neonatal complications (9–11). Numerous individual studies and meta-analyses have addressed the association between CA and complications of very/extremely preterm birth such as bronchopulmonary dysplasia (12, 13), necrotizing enterocolitis (14), retinopathy of prematurity (15, 16), patent ductus arteriosus (17, 18), intraventricular hemorrhage (19), cerebellar hemorrhage (20), neonatal brain injury (21), or cerebral palsy (22).

Very/extremely preterm infants are at high risk for neonatal sepsis (23–27). Early onset sepsis (EOS) is defined as a blood or cerebrospinal fluid culture obtained within 72 h after birth. EOS is typically caused by microorganisms transmitted vertically from the mother to the infant before birth or during delivery (23, 24). Frequently, preterm EOS begins in the uterus and the microbial-induced maternal inflammation initiates labor and elicits an inflammatory response in the fetus (23–25). Therefore, CA and/or intraamniotic infection are strongly associated with EOS in preterm infants (23–25).

Late-onset sepsis (LOS) occurs after 72 h of life and may be caused by microorganisms acquired at delivery or during the course of hospital care (23, 25). Very/extremely preterm infants are at an increased risk of LOS because of the relative immaturity of their immune system as well as the frequently required prolonged hospitalization, with ongoing risk of infection, and exposure to invasive procedures and devices (23, 25, 26, 28). Coagulase-negative staphylococci (CoNS) are the most frequent pathogens causing nosocomial sepsis among preterm infants. Interestingly, Strunk et al. reported in a cohort of infants with a GA below 30 weeks that histological CA was associated with reduced risk of developing LOS, both with CoNS and other bacteria (25). They speculate that “chorioamnionitis may result in maturation of the fetal and neonatal immune system and therefore indirectly modulates the risk of LOS with nosocomial organisms” (25).

Surprisingly, to the best of our knowledge, the association between CA and neonatal sepsis has not yet been the subject of a systematic review. We therefore aimed to carry out a systematic review and meta-analysis of observational studies reporting on the association between CA and EOS and/or LOS in preterm infants. We paid particular attention to how the criteria used to define CA and sepsis affected the potential association between the two conditions. We also analyzed the role of potential confounders or intermediate factors, such as GA, birth weight (BW), presence of fetal inflammatory response (i.e., funisitis), or exposure to antenatal corticosteroids, on the association between CA and neonatal sepsis.

## Methods

We used a similar methodology to earlier meta-analyses on the association of CA and short-term outcomes of prematurity (13, 16, 17, 19). A protocol (available at https://www.crd.york.ac.uk/prospero/display_record.php?RecordID=117190) was developed and published a priori in which the objectives, inclusion criteria, method for evaluating study quality, included outcomes and covariates, and statistical methodology were specified (29) We report the study according to the guidelines for the Preferred Reporting Items for Systematic Reviews and Meta-Analysis (PRISMA) (30).

### Sources and Search Strategy

A comprehensive literature search was performed in the PubMed/MEDLINE and EMBASE databases from their inception to December 1, 2018. The search strategy involved the following keywords in various combinations: “chorioamnionitis,” “intrauterine infection,” “intrauterine inflammation,” “antenatal infection,” antenatal inflammation,” “funisitis,” “preterm infant,” “prematurity,” “cohort,” “sepsis,” “(neonatal) early-onset sepsis,” and “(neonatal) late-onset sepsis.” The full search strategy can be found in the Supplementary Data 1.

### Study Selection

Studies were included if they examined preterm (GA <37 weeks) infants and reported primary data that could be used to measure the association between exposure to CA and the development of neonatal sepsis. Studies using BW instead of GA as the inclusion criteria were included only when inclusion BW was below 1,500 g. Therefore, we selected studies assessing the outcomes of infants exposed to CA when sepsis was one of the reported outcomes, and studies assessing the risk factors for sepsis when CA was one of the reported risk factors. We also included studies reporting on intra-amniotic infection resulting from microbial invasion of the amniotic cavity (MIAC) because MIAC is also within the spectrum of CA (31). MIAC was defined as a positive amniotic fluid culture for microorganisms. The results of the total search were screened independently by two reviewers (G. A. L. and O. M. R.) in several rounds: first by title only, second by title and abstract and thirdly by consulting the full text. The reviewers resolved discrepancies in inclusion through discussion and by consulting a third reviewer (P. D.).

### Data Extraction

Utilizing a predetermined worksheet, data was extracted from the included studies by three researchers (G. A. L., O. M. R., and E. V.-M). Two additional researchers (P. D. and E. V.) checked the extracted data for accuracy and completeness. Discrepancies were resolved by checking the primary data report and by discussion. We extracted the following data from each study: citation information, the language of the publication, the location where research was conducted, the time period of the study, study objectives, study design, inclusion/exclusion criteria, patient characteristics, definitions of CA (clinical, histological, or microbiological), definitions of sepsis (culture-proven or clinical), and results (including raw numbers, summary statistics, and adjusted analyses on CA and sepsis where available). Onset of sepsis was classified in three groups: EOS, LOS, and unspecified onset sepsis (UOS).

### Quality Assessment

The Newcastle-Ottawa Scale (NOS) for cohort or case-control studies was used to assess the methodological quality of the included studies (32). The NOS evaluates three aspects of a given study: selection, comparability, and exposure/outcome. These are scored individually and tallied up to a possible total of 9 points. The NOS was independently used by two researchers (G. A. L. and E. V.-M) to evaluate the quality of each study. Discrepancies were resolved by reaching consensus through discussion.

### Statistical Analysis

Studies were combined and analyzed using COMPREHENSIVE META-ANALYSIS V 3.0 software (CMA, RRID:SCR_012779, Biostat Inc., Englewood, NJ, USA). The odds ratio (OR) and the 95% confidence interval (CI) for dichotomous variables were calculated from the extracted data of the studies. For continuous variables, the mean difference (MD) was calculated together with the 95% CI. When studies reported continuous variables as the median and the range/interquartile range, we estimated the mean and standard deviation using the method of Wan et al. (33). Due to anticipated heterogeneity, summary statistics were calculated with a random-effects model. This accounts for the variability between studies as well as within studies. For subgroup analyses the mixed-effects model was used (34). With this approach, a random-effects model is used within each subgroup, while a fixed-effect model is used to combine subgroups to generate the overall effect. The study-to-study variance (tau-squared) was calculated across all studies. Statistical heterogeneity was tested using Cochran's Q statistic and the *I*^2^ statistic (34). Publication bias was assessed with Egger's regression test (35) and visual inspection of funnel plots. K represents the number of studies used in each analysis.

Univariate random-effects meta-regression was used to explore whether the differences in covariates between studies might influence the outcome effect size (34). We carried out meta-regression analysis only if there were more than 10 studies that reported on a covariate. The following possible sources of variability were defined beforehand: CA type (clinical or histological), sepsis type (culture-proven or clinical), differences in GA and BW between infants with and without CA, use of antenatal corticosteroids, mode of delivery, rate of GA, rate of PROM, and rate of preeclampsia.

## Results

### Description of Studies

**Figure 1:**
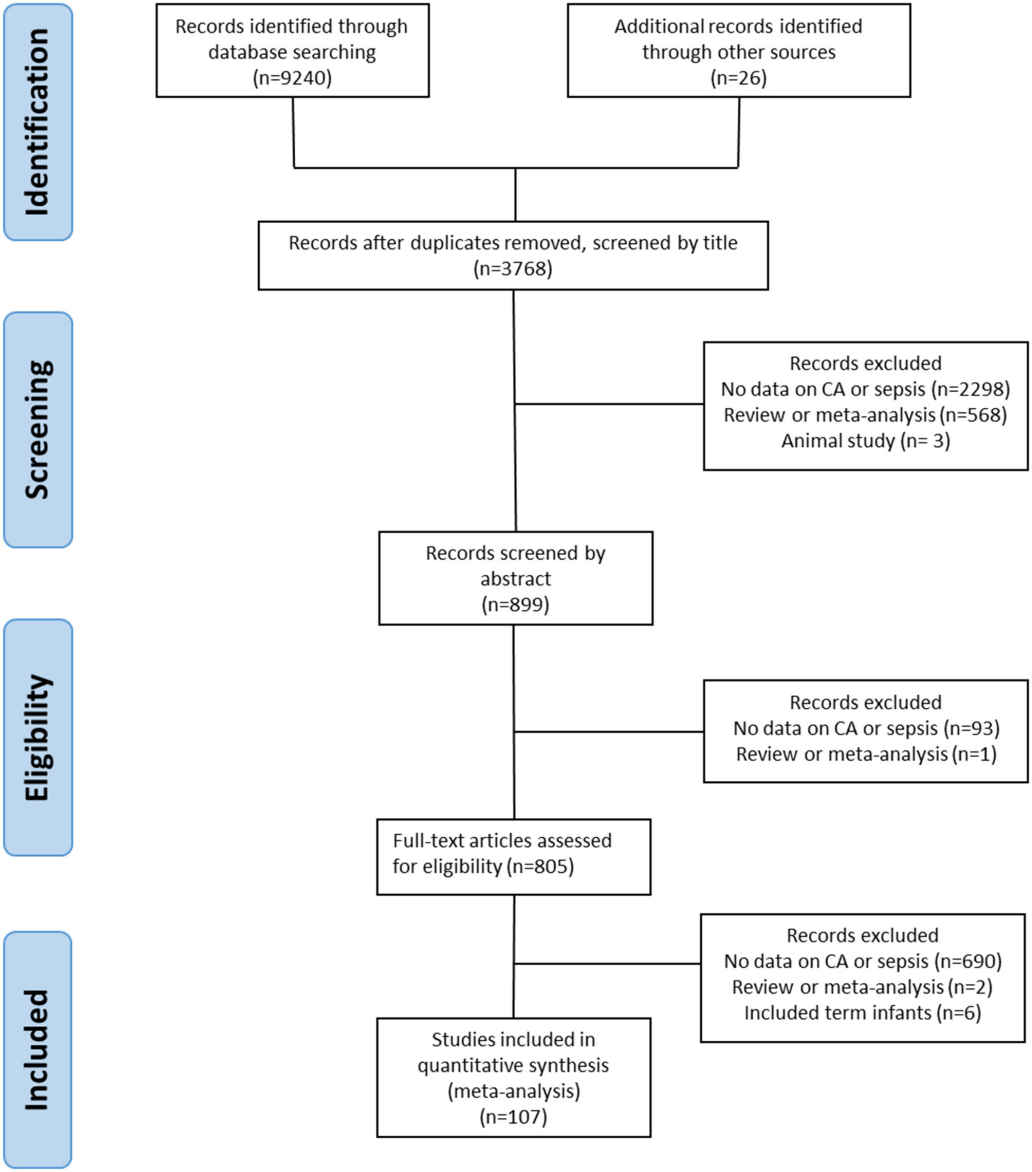
PRISMA diagram of the systematic search.

We screened 3,768 studies after removing duplicates, of which 107 studies met the inclusion criteria (4, 25, 36–141). The PRISMA flow diagram of the search is shown in Figure 1. The included studies evaluated 387,321 infants, including 44,414 infants with CA. An overview of the characteristics of the included studies can be found in Supplementary Table 1. There were 75 studies that evaluated the outcomes of CA, and sepsis was one of these outcomes, and 26 studies that looked at potential risk factors for sepsis, including CA. Seven studies were designed to primarily examine the association between CA and sepsis. From the included studies, 58 used a histological definition of CA and 28 studies used a clinical definition of CA. Nine studies distinguished between both definitions of CA in their reporting (36, 45, 65, 80, 93, 97, 104, 106, 108). Five studies reported on MIAC (77, 83, 84, 105, 113). In six studies, the CA definition was not further specified and categorized as “unspecified” for further analysis (37, 42, 75, 103, 129). Nine studies (37, 46, 77, 98, 115, 118, 129, 139, 141) used the 7-day limit to differentiate between EOS and LOS.

### Quality Assessment

A summary of the NOS quality assessment can be found in Supplementary Table 2. Four studies received a quality score of 5 points, 24 studies a score of 6 points, 57 studies a score of 7 points, 9 studies a score of 8 points and 13 studies received a score 9 points. Studies lost points for quality for not adjusting the risk of sepsis for confounders (*k* = 90), for not defining sepsis clearly (*k* = 6), for not defining CA clearly (*k* = 21), and for adjusting the risk of sepsis only for one confounder (*k* = 6).

### Meta-Analysis Based on Unadjusted Data

#### Early Onset Sepsis

**Figure 2:**
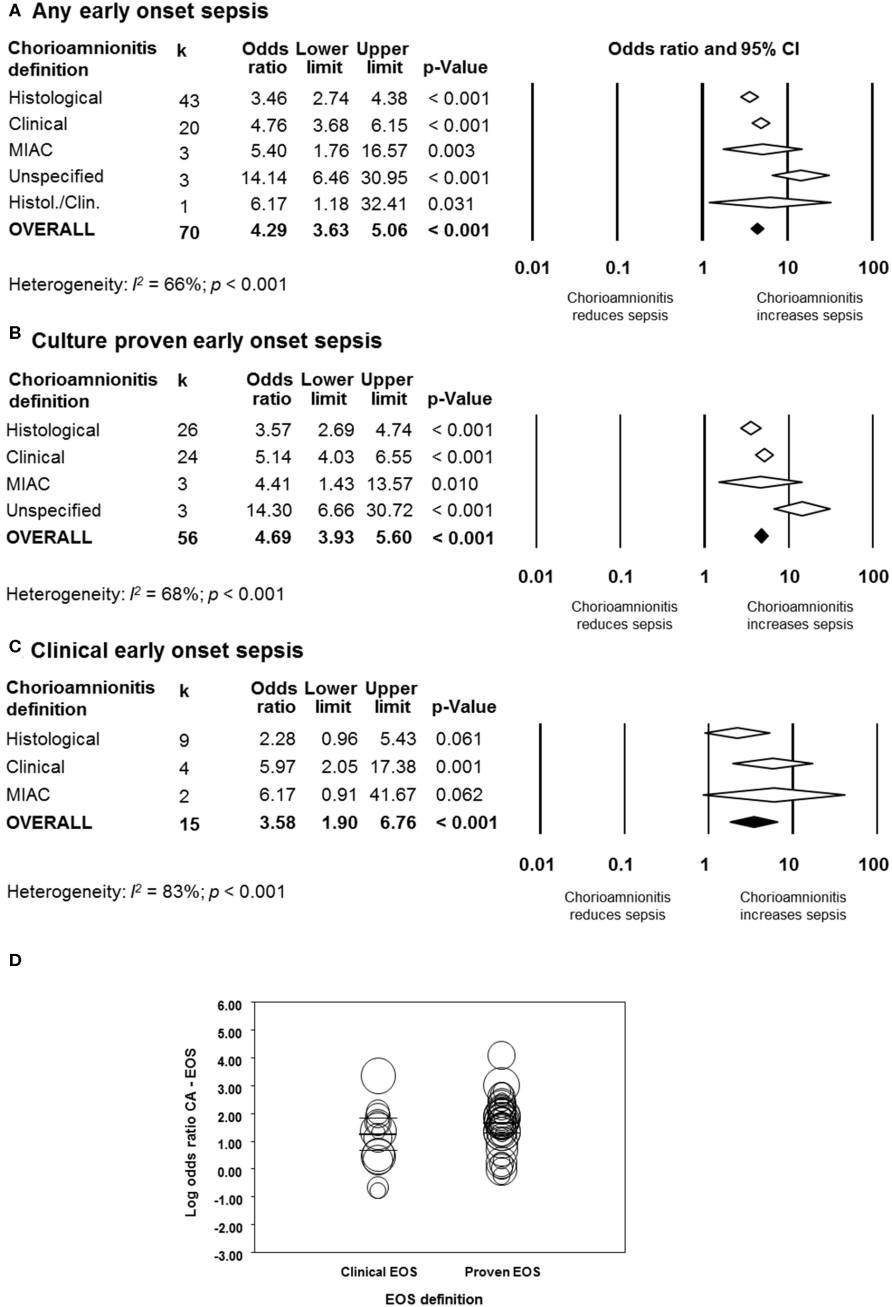
Random effects meta-analyses of chorioamnionitis and early onset sepsis (EOS), subdivided by definition of chorioamnionitis. **(A)** Any EOS; **(B)** culture-proven EOS; **(C)** clinical EOS; **(D)** meta-regression comparing culture-proven and clinical EOS. MIAC, microbial invasion of the amniotic cavity.

**Figure 3:**
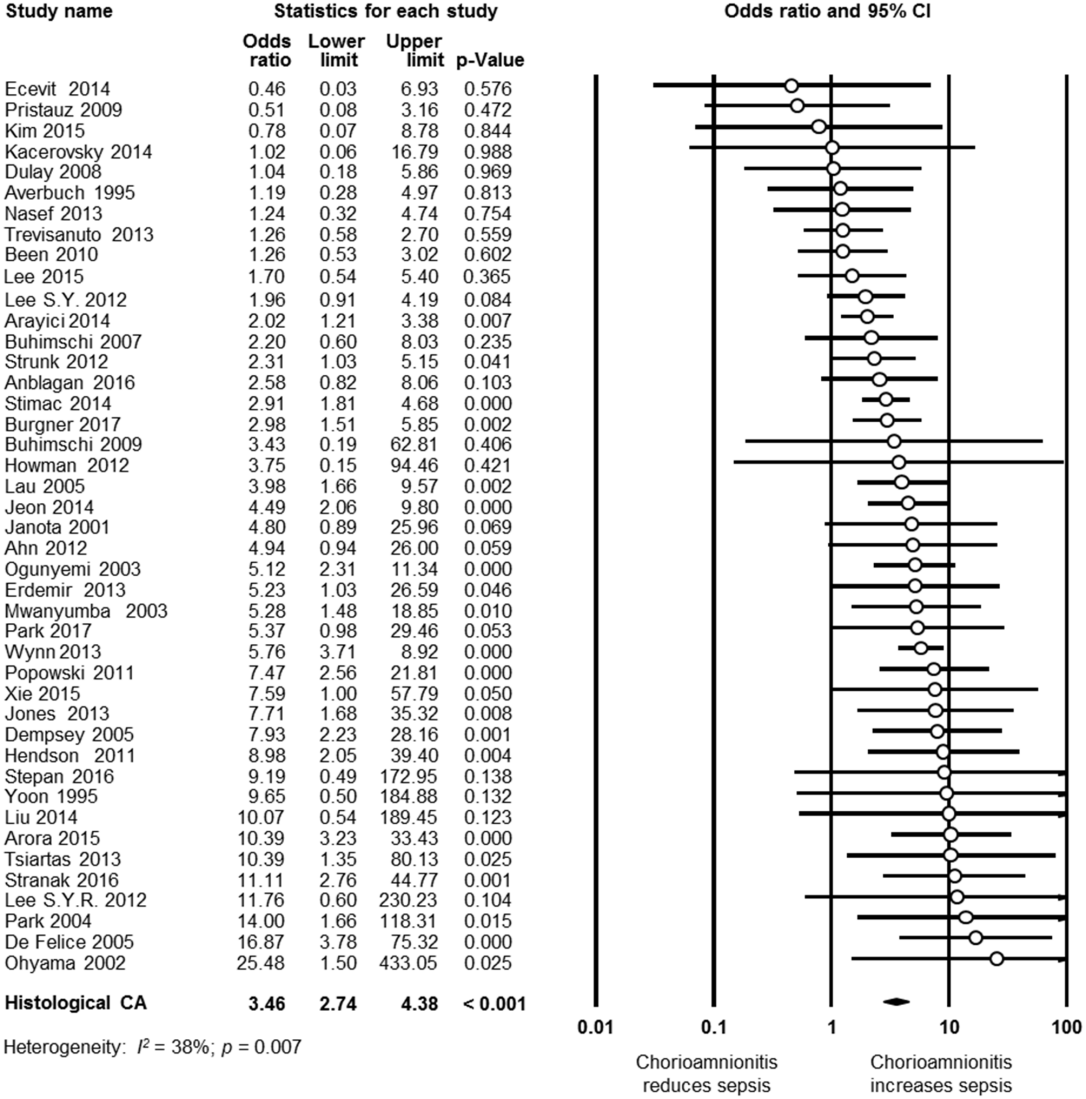
Random effects meta-analysis of histological chorioamnionitis (CA) and all early onset sepsis (culture proven or clinical).

**Figure 4:**
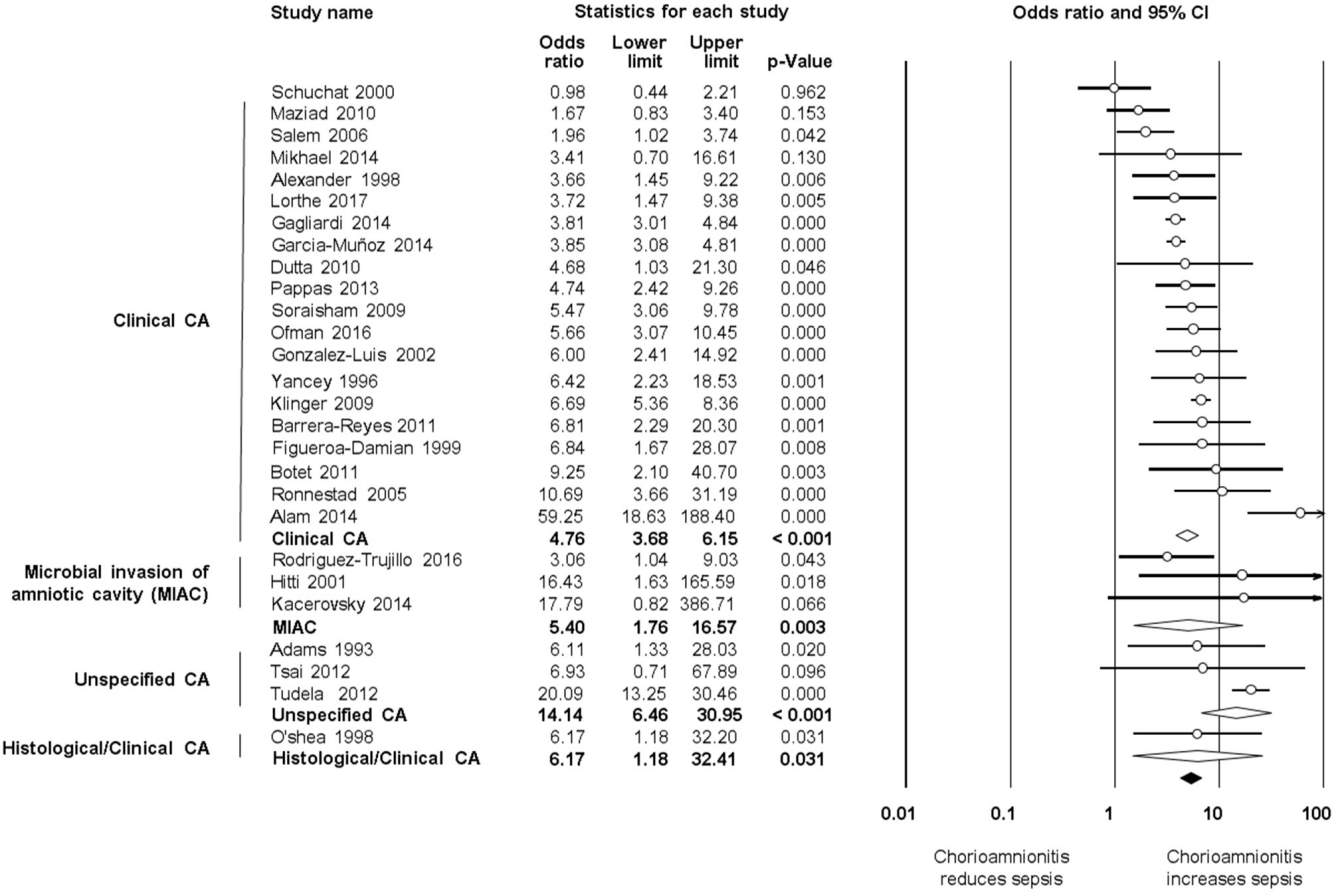
Random effects meta-analyses of clinical, microbiological, unspecified, and histological/clinical chorioamnionitis (CA) and all early onset sepsis (culture proven or clinical).

As shown in Figure 2A, meta-analysis found a positive association between CA (any type) and EOS (any type) (*k* = 70, OR 4.29, 95% CI 3.63–5.06). When subdividing by CA definition, meta-analysis showed that histological CA (*k* = 43, OR 3.46, 95% CI 2.74–4.4.38, Figure 3), clinical CA (*k* = 20, OR 4.76, 95% CI 3.68–6.15, Figure 4), histological/clinical CA (*k* = 1, OR 6.17, 95% CI 1.18–32.23, Figure 4), MIAC (*k* = 3, OR 5.38, 95% CI 1.76–16.44, Figure 4), and unspecified CA (*k* = 3, OR 14.14, 95% CI 6.46–30.95, Figure 4) were associated with EOS (any type). The exclusion of the studies reporting on MIAC, combined clinical/histological CA, and unspecified CA did not substantially affect the OR of the association between CA and EOS (Table 1).

**Table 1:** Meta-analysis on the association between any chorioamnionitis^a^ and sepsis, divided by onset, and definition of sepsis.

**Sepsis onset**	**Sepsis definition**	**Number of studies (k)**			**OR**	**95% CI**	***P***
		**Total**	**Histological**	**Clinical**			
Early	Any sepsis	64	42	22	4.10	3.45–4.86	<0.001
	Culture proven	50	26	24	4.40	3.66–5.29	<0.001
	Clinical	13	9	4	3.34	1.71–6.53	<0.001
Late	Any sepsis	27	21	6	1.31	1.12–1.53	0.001
	Culture proven	16	12	4	1.33	1.13–1.57	0.001
	Clinical	5	5	0	1.52	0.78–2.96	0.216
Unspecified	Any sepsis	29	22	7	1.61	1.30 - 1.99	<0.001
	Culture proven	20	15	5	1.48	1.16 - 2.27	<0.001
	Clinical	6	5	1	2.13	1.30 - 3.48	0.003

a *Studies reporting on microbial invasion of the amniotic cavity (MIAC), combined clinical/histological chorioamnionitis, or on unspecified chorioamnionitis were excluded from the analysis*.

When subdividing by EOS definition, meta-analysis showed that CA (any type) was associated with culture-proven EOS (*k* = 56, OR 4.69, 95% CI 3.93–5.60, Figure 2B) and clinical EOS (*k* = 15, OR 3.58, 95% CI 1.90–6.76, Figure 2C). As shown in Figure 2B, subgroup analysis based on CA type showed that histological CA (*k* = 26, Supplementary Figure 1), clinical CA (*k* = 24 Supplementary Figure 2), MIAC (*k* = 3, Supplementary Figure **2**), and unspecified CA (*k* = 3, Supplementary Figure 2) were associated with culture-proven EOS. In contrast, as shown in Figure 2C and Supplementary Figure 3, clinical EOS was associated with clinical CA (*k* = 4), but not with histological CA (*k* = 9), or MIAC (*k* = 2). Meta-regression could not find differences between the effect size of the association CA-culture-proven EOS and the effect size of the association CA-clinical EOS (*p* = 0.150, Figure 2D). There was no evidence of publication bias for studies reporting on EOS (Supplementary Figure 4), assessed with Egger's regression test and visual inspection of the funnel plots.

#### Late Onset Sepsis

**Figure 5:**
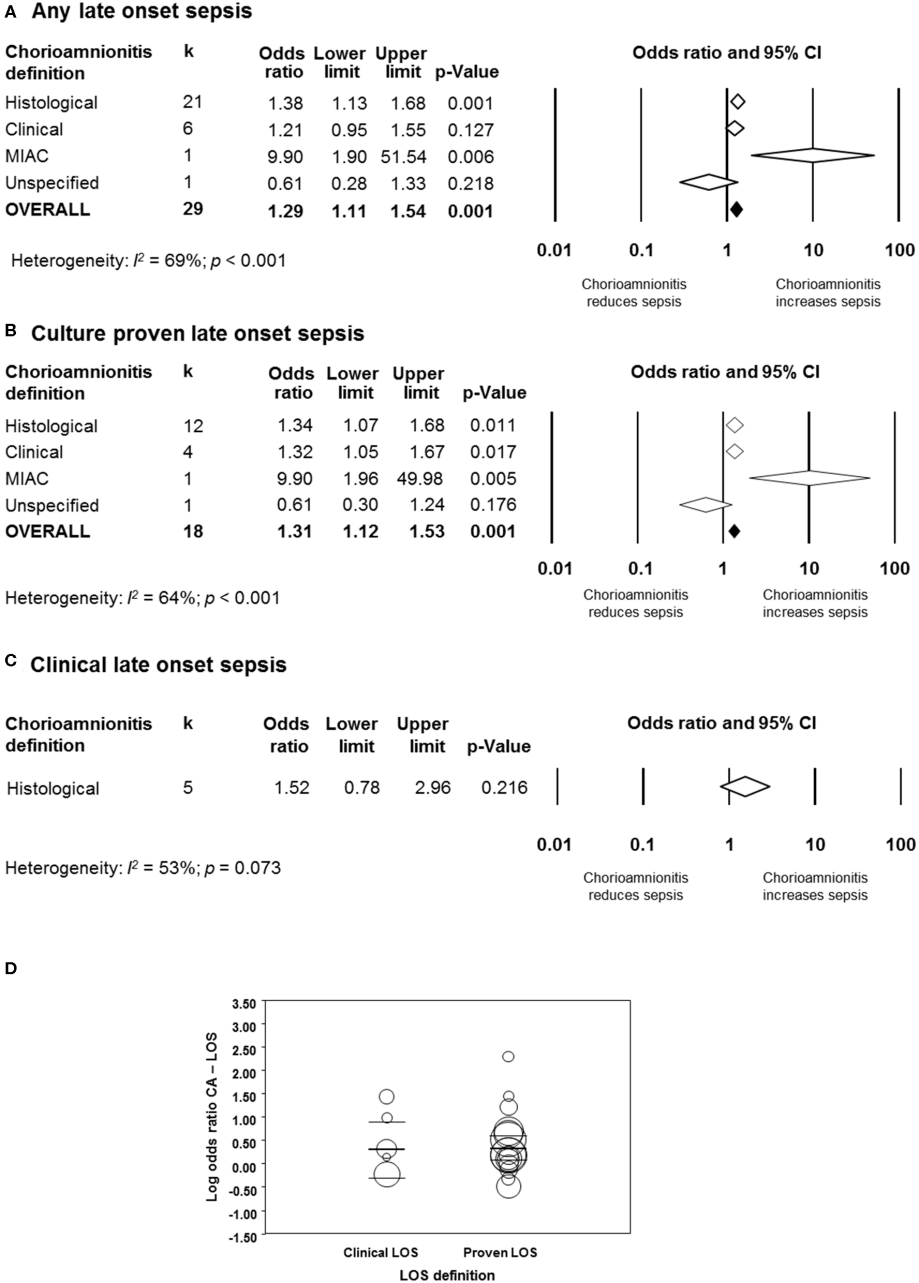
Random effects meta-analyses of chorioamnionitis and late onset sepsis (LOS), subdivided by definition of chorioamnionitis. **(A)** Any LOS; **(B)** culture-proven LOS; **(C)** clinical LOS; **(D)** meta-regression comparing culture-proven and clinical LOS. MIAC, microbial invasion of the amniotic cavity.

**Figure 6:**
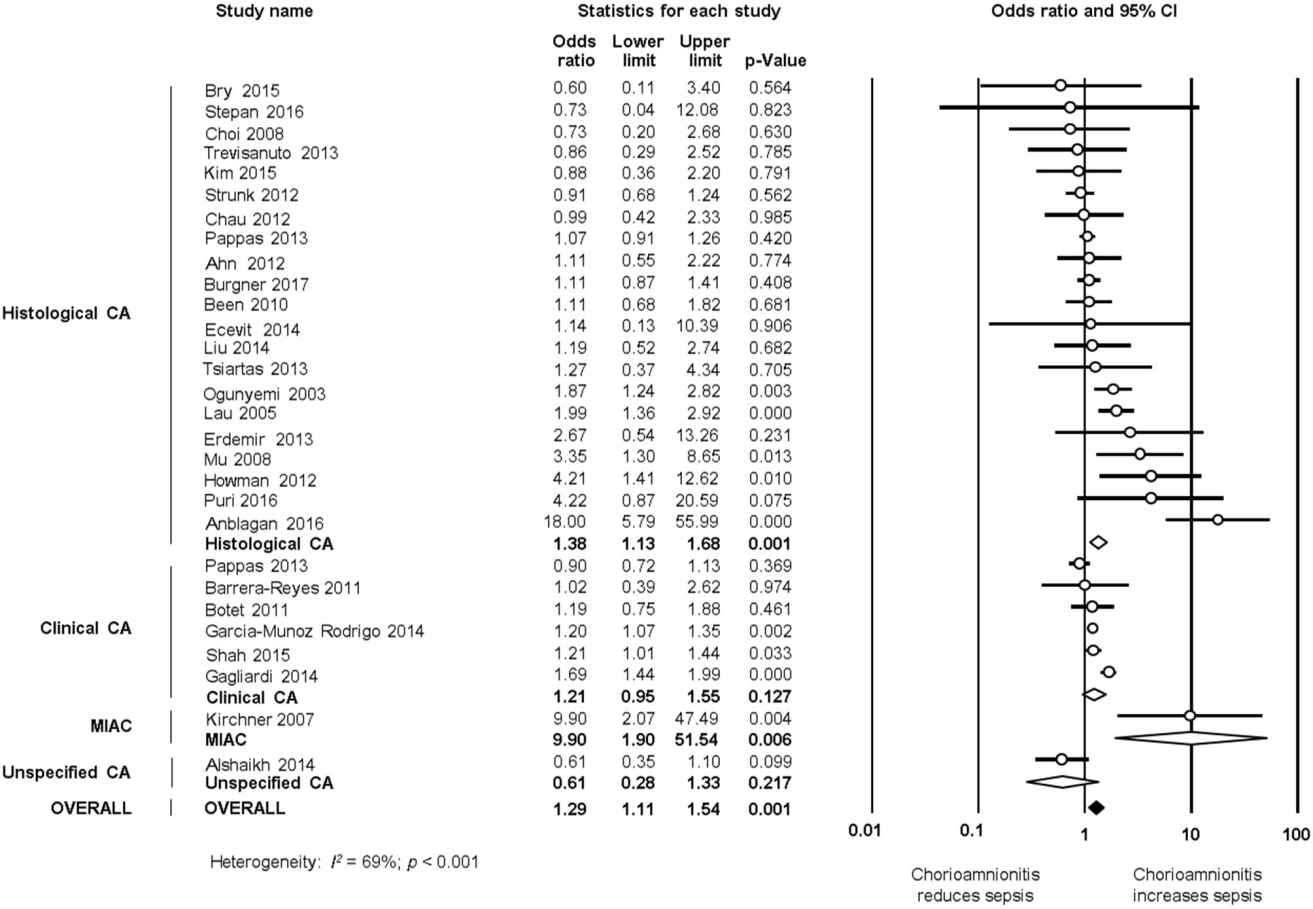
Random effects meta-analyses of histological, clinical, microbiological, and unspecified chorioamnionitis (CA) and all late onset sepsis.

As shown in Figures 5A, 6, meta-analysis found a positive association between CA (any type) and LOS (any type) (*k* = 29, OR 1.29, 95% CI 1.11–1.54). When subdividing by CA definition, meta-analysis showed that histological CA (*k* = 21, OR 1.38, 95% CI 1.13–1.68, Figure 6), and MIAC (*k* = 1, OR 9.90, 95% CI 1.90–51.54, Figure 6) were associated with LOS (any type). In contrast, subgroup analysis could not find an association between LOS (any type) and clinical CA (*k* = 6, OR 1.21, 95% CI 0.95–1.55, Figure 6), or unspecified CA (*k* = 1 OR 0.61, 95% CI 0.28–1.33, Figure 6). The exclusion of the studies reporting on MIAC, combined clinical/histological CA, and unspecified CA did not substantially affect the OR of the association between CA and LOS (Table 1).

When subdividing by LOS definition, meta-analysis showed that CA (any type) was associated with culture-proven LOS (*k* = 18, OR 1.31, 95% CI 1.12–1.53, Figure 5B and Supplementary Figure 5), but not with clinical LOS (*k* = 5, OR 1.52, 95% CI 0.78–2.96, Figure 5C and Supplementary Figure 6). The analysis on the association between clinical LOS and CA was exclusively based on data on histological CA. As shown in Figure 5B and Supplementary Figure 5, subgroup analysis based on CA type showed that histological CA (*k* = 12), clinical CA (*k* = 4), and MIAC (*k* = 1) were associated with culture-proven LOS. Meta-regression could not find differences between the effect size of the association CA-culture-proven LOS and the effect size of the association CA-clinical LOS (*p* = 0.920, Figure 5D). There was no evidence of publication bias for studies reporting on LOS (Supplementary Figure 4), assessed with Egger's regression test and visual inspection of funnel plots.

#### Unspecified Onset Sepsis

**Figure 7:**
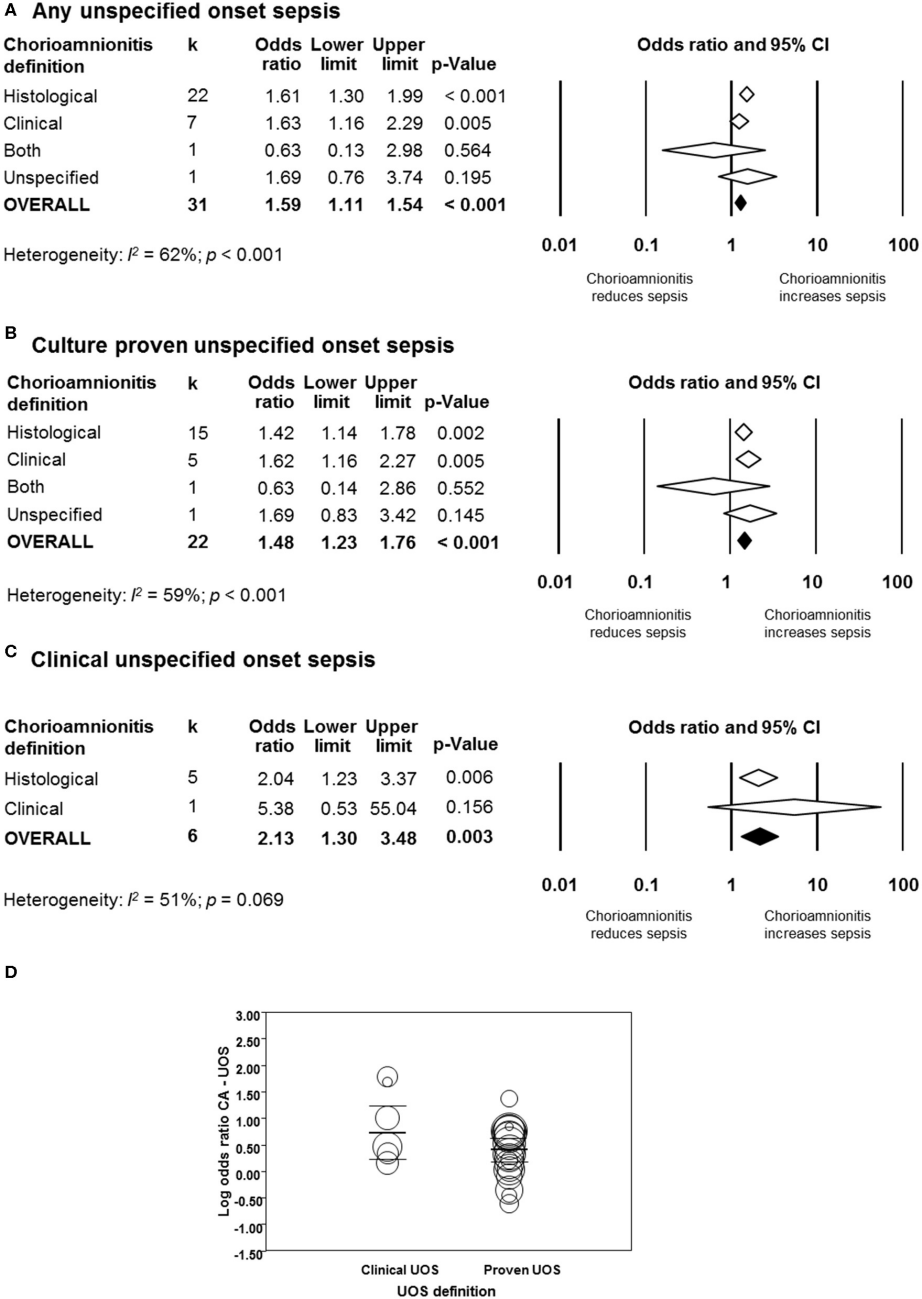
Random effects meta-analyses of unspecified onset sepsis (UOS), subdivided by definition of chorioamnionitis. **(A)** Any UOS; **(B)** culture-proven UOS; **(C)** clinical UOS; **(D)** meta-regression comparing culture-proven and clinical UOS.

As shown in Figure 7A and Supplementary Figure 7, meta-analysis found a significant positive association between CA (any type) and UOS (any type) (*k* = 31, OR 1.59, 95% CI 1.11–1.54). When subdividing by CA definition, meta-analysis showed that histological CA (*k* = 22, OR 1.61, 95% CI 1.30–1.99), and clinical CA (*k* = 7, OR 1.63, 95% CI 1.16–2.29) were significantly associated with UOS (any type, Supplementary Figure 7).

When subdividing by UOS definition, meta-analysis showed that CA (any type) was associated with culture-proven UOS (*k* = 22, OR 1.48, 95% CI 1.23–1.76, Figure 7B and Supplementary Figure 8) and clinical UOS (*k* = 6, OR 2.13, 95% CI 1.30–3.48, Figure 7C and Supplementary Figure 9). As shown in Figure 7B and Supplementary Figure 8, subgroup analysis based on CA type showed that histological CA (*k* = 15), and clinical CA (*k* = 5) were associated with culture-proven UOS. In contrast, only histological CA was associated with clinical UOS (*k* = 5, Figure 7C and Supplementary Figure 9). Meta-regression could not find differences between the effect size of the association CA-culture proven UOS and the effect size of the association CA-clinical UOS (Figure 7D). There was no evidence of publication bias for studies reporting on UOS (Supplementary Figure 4), assessed with Egger's regression test and visual inspection of funnel plots.

#### Funisitis

**Figure 8:**
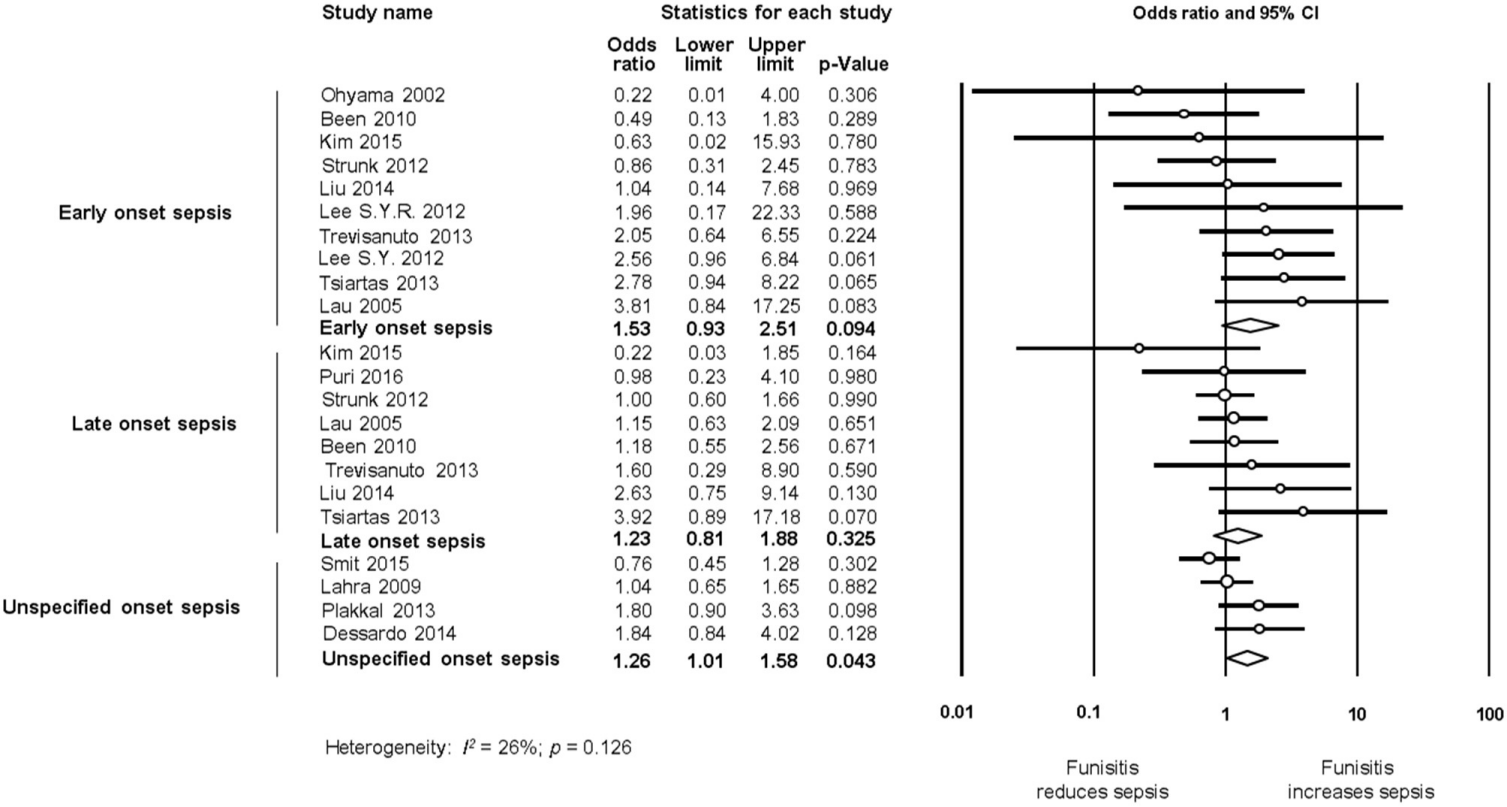
Random effects meta-analyses of funisitis (compared with chorioamnionitis without funisitis) and sepsis (culture proven or clinical), subdivided by onset of sepsis.

Additional meta-analyses were performed to determine if funisitis, as a fetal-inflammatory response, was associated with the development of neonatal sepsis. As shown in Figure 8, 10 studies reported on EOS and infants with histological CA with or without funisitis; eight studies on LOS and infants with histological CA with or without funisitis; and five studies on UOS and infants with histological CA with or without funisitis. Meta-analysis showed that funisitis did not increase the risk of sepsis (EOS, LOS, or UOS), when compared with CA without funisitis (Figure 8).

### Meta-Analysis of Covariates and Meta-Regression

We performed additional meta-analyses to explore the possible differences in baseline characteristics between the groups exposed and non-exposed to CA. Exposure to CA was significantly associated with lower GA and BW, as shown in Table 2. Additionally, when compared with CA-unexposed, CA-exposed infants had significantly higher rates of exposure to antenatal corticosteroids and PROM, and significantly increased rates of mortality, but significantly lower rates of preeclampsia, cesarean section, or small for GA (Table 3).

**Table 2:** Meta-analysis of chorioamnionitis and continuous variables.

**Meta-analysis**	**CA Type**	***K***	**Mean difference**	**95% CI**	***Z***	***p***	**Heterogeneity**		
							***Q***	***p***	***I*^**2**^(%)**
Gestational age (weeks)	Clinical	13	−0.84	−1.32 to −0.36	−20.8	0.000	1358.6	0.000	99.1
	Histological	47	−1.11	−1.37 to −0.84	−20.8	0.000	888.5	0.000	94.8
	Microbiological	5	−2.23	−3.10 to −1.36	−14.8	0.000	160.4	0.000	97.5
	Any Type	67	−1.11	−1.34 to −0.89	−70.8	0.000	2961.5	0.000	97.8
Birth weight (g)	Clinical	12	−19.12	−83.9 to 45.6	−1.3	0.563	490.5	0.000	97.8
	Histological	47	−49.96	−73.6 to −26.1	−64.9	0.000	255.9	0.000	82.0
	Microbiological	4	−226.8	−434.5 to −19.14	−4.93	0.032	12.1	0.007	75.2
	Any Type	65	−48.30	−70.25 to −26.35	−62.6	0.000	1078.8	0.000	94.0
Maternal age (years)	Clinical	6	0.62	−0.50 to 1.76	−10.9	0.280	59.4	0.000	91.6
	Histological	25	−0.11	−0.48 to 0.27	−2.2	0.580	40.3	0.027	40.5
	Any Type	33	−0.01	−0.34 to 0.33	−10.4	0.976	116.1	0.000	72.4

*CA, chorioamnionitis; k, number of studies included. Mean difference represent the mean of the CA-exposed group minus the mean of the CA-unexposed group*.

**Table 3:** Meta-analysis of chorioamnionitis and dichotomous variables.

**Meta-analysis**	**CA Type**	***k***	**OR**	**95% CI**	***Z***	***P***	**Heterogeneity**		
							***Q***	***p***	***I*^2^(%)**
Diabetes	Any Type	8	0.20	0.01 to 4.38	−1.5	0.310	3.4	0.844	0.0
Antenatal corticosteroids^a^	Clinical	6	1.54	1.38 to 1.71	7.2	0.000	181.3	0.000	97.8
	Histological	28	1.36	1.25 to 1.48	7.9	0.000	103.8	0.000	75.9
	Any Type	37	1.42	1.33 to 1.52	10.3	0.000	262.9	0.000	86.3
Pre-eclampsia	Histological	14	0.16	0.10 to 0.27	−17.4	0.000	47.7	0.000	72.7
	Any Type	16	0.09	0.09 to 0.21	−18.2	0.000	61.5	0.000	75.6
Preterm rupture of membranes	Clinical	7	3.80	2.27 to 6.38	37.2	0.000	111.3	1.000	94.6
	Histological	28	2.74	2.18 to 3.44	22.4	0.000	102.3	0.000	73.6
	Any Type	36	2.84	2.32 to 3.47	43.4	0.000	220.4	0.000	0.45
Cesarean section	Clinical	11	0.55	0.20 to 1.57	22.5	0.267	2721.7	0.000	99.6
	Histological	26	0.40	0.30 to 0.52	−18.9	0.000	140.1	0.000	82.1
	Any Type	39	0.43	0.34 to 0.55	10.7	0.000	3613.7	0.000	98.9
Small for gestational age	Clinical	3	0.33	0.16 to 0.66	−5.3	0.002	4.8	0.089	58.7
	Histological	16	0.36	0.23 to 0.55	−13.7	0.000	70.6	0.000	78.7
	Any Type	19	0.35	0.14 to 0.81	−5.3	0.000	76.1	0.000	76.2
Mortality	Clinical	9	1.64	1.34 to 2.01	1442.1	0.000	23.5	0.003	65.9
	Histological	32	1.54	1.28 to 1.85	6.1	0.000	51.0	0.013	39.3
	Any Type	44	1.59	1.39 to 1.82	13.3	0.000	84.1	0.000	48.9

*CA, chorioamnionitis; k, number of studies included; OR, odds ratio. An OR higher than 1 indicates that CA is a risk factor for the outcome, an OR below indicates that CA is a protective factor for the outcome*.

a *Complete or partial course*.

**Figure 9:**
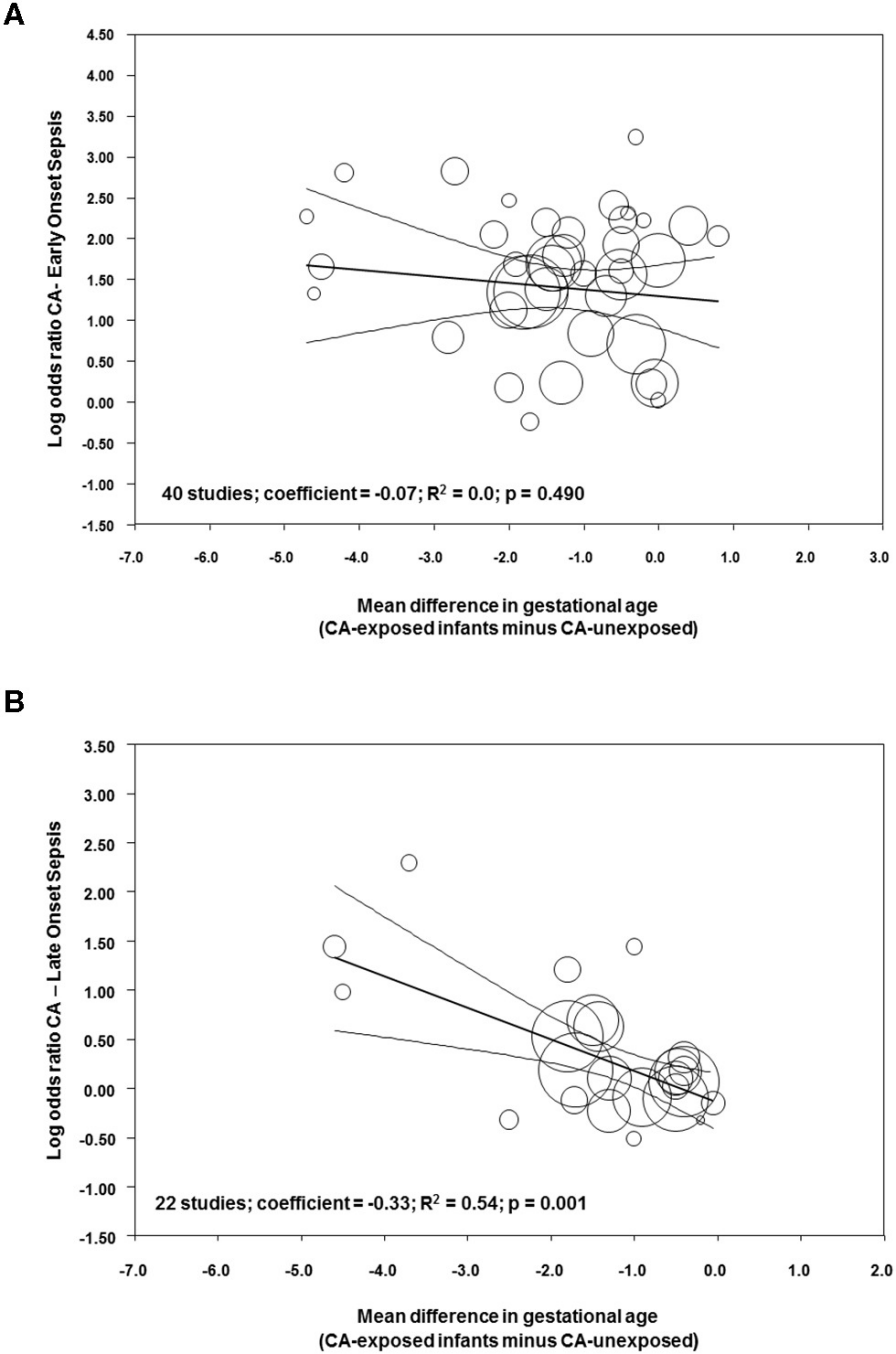
Meta-regression plot of association between chorioamnionitis and **(A)** early onset sepsis, and **(B)** late onset sepsis controlling for difference in gestational age between exposed and non-exposed groups.

Meta-regression was performed to determine the potential modulatory effect of GA and BW on the association between sepsis and CA. As shown in Figure 9A, the effect size of the association between CA and EOS was not affected by the differences in GA between the CA-exposed and CA-unexposed group (*R*^2^ = 0.0; *p* = 0.490). In contrast, meta-regression showed that mean differences in GA modified the effect size of the association between CA and LOS (*R*^2^ = 0.54, *p* = 0.001, Figure 9B). Meta-regression for mean differences in BW could not show a correlation with the effect size of the association CA-EOS (Supplementary Figure 10A) or with the effect size of the association CA-LOS (Supplementary Figure 10B). Meta-regressions of the associations CA-EOS and CA-LOS with other covariates (antenatal corticosteroids, cesarean section, small for GA, PROM) did not show correlations with these covariates (Supplementary Table 2).

### Meta-Analysis Subdivided by Gestational Age

To further assess the effect of GA on the association CA-sepsis, we pooled the studies where the mean difference in GA was, according to the classical threshold of *p*-value (*p* ≥ 0.05) (142), “non-significant” and compared it with meta-analysis of studies where the mean difference in GA was “statistically significant” (*p* < 0.05). Meta-analysis found that CA was a risk factor for EOS both in studies where infants had a similar GA across groups (*k* = 15, OR 3.76, 95% CI 2.66–5.32, Supplementary Figure 11), and in studies where infants had significantly lower GA in the CA group (*k* = 22, OR 3.95, 95% CI 3.11–5.02, Supplementary Figure 11). In contrast, the association between CA and LOS was only observed in the subgroup meta-analysis of studies where CA-exposed infants had lower GA (*k* = 16, OR 1.33, 95% CI 1.11–1.60, Supplementary Figure 12), but not in the subgroup of studies where infants had similar GA across groups (*k* = 7, OR 1.06, 95% CI 0.72–1.57, Supplementary Figure 12).

### Meta-Analysis Based on Adjusted Data

We pooled studies that provided adjusted data on the association between CA and sepsis. Meta-analysis of adjusted data found an association between CA and EOS (*k* = 15, OR 2.51, 95% CI 1.51–4.14, Supplementary Figure 13), but not between CA and LOS (*k* = 7, OR 1.04, 95% CI 0.79–1.38, Supplementary Figure 14). Meta-analysis of unadjusted data of the same group of studies found that CA was associated with both EOS (OR 5.10, 95% CI 3.07–8.46, Supplementary Figure 13) and LOS (OR 1.42, 95% CI 1.08–1.87, Supplementary Figure 14).

## Discussion

This is the first meta-analysis investigating the association between CA and sepsis in preterm infants. The meta-analysis based on unadjusted data showed a strong association between CA and EOS and a weaker, but still positive, association between CA and LOS. Exposure to funisitis was not associated with a higher risk of sepsis when compared with exposure to CA in the absence of funisitis. As in previous meta-analysis on the association between CA and short term outcomes of prematurity (12, 13, 16, 17, 19), we observed differences in basal characteristics between the CA-exposed and the CA-unexposed group. CA was associated with a lower GA and BW, higher rates of exposure to antenatal corticosteroids, and the preterm rupture of membranes, as well as lower rates of cesarean section and small for gestational age. Meta-regression analysis showed that the lower GA of the CA group correlated with the association between CA and LOS but not with the association between CA and EOS. Therefore, the pathogenic effect of CA on LOS appears to be modulated by the effect of chorioamnionitis on GA.

We chose to combine studies by using ORs rather than risk ratios (RRs) because it allowed us to compare the unadjusted ORs with the adjusted ORs reported in some studies. However, while the RR has a relatively simple interpretation, OR interpretation is less intuitive, as the concept of “odds” is less easy to grasp (143, 144). Interpretation of ORs in the same way as RRs can lead to overestimation of effect sizes when the risk in either group is high (above 20%) and the OR is large (144). In order to check if there were important discrepancies between ORs and RRs in our study, we calculated the RRs for some analyses. In the case of the association between CA and EOS, the RR was 3.45, whereas the OR was 4.29 (~24% higher). In the case of the association between CA and LOS, the RR was 1.21 whereas the OR was 1.29 (~6.6% higher). Therefore, in CA-exposed infants, the risk of EOS was 3.5-fold higher and the risk of LOS was 1.2-fold higher than the respective risk in infants non-exposed to CA. These increases in risk were slightly overestimated by the OR.

Since very/extremely preterm birth is always a pathological condition, any study aimed to analyze the association between its etiology and its outcome will face the limitation of the absence of a healthy control group (145). As mentioned in the introduction, very/extremely preterm birth etiology can be divided into two main categories: intrauterine infection/inflammation and placental vascular dysfunction (2–4). The distribution of these two etiological entities across the different gestational ages is not uniform: the lower the GA, the greater the possibility that an infectious/inflammatory process is the trigger for preterm birth (2, 146). Our meta-analysis showed that infants exposed to CA were born ~1.1 weeks earlier than “control” infants. Meta-regression showed that this difference in GA did not affect the association between CA and EOS but modulated the association between CA and LOS. The difference in GA was associated with 54% (*R*^2^) of the variance in the association between CA and LOS across studies and each week that infants with CA were born earlier than control infants resulted in an increase in LOS log OR of 0.33 (the equivalent of going from an OR of 1.00 to an OR of 2.14). This modulatory role of GA may be related to the maturation of the immunological system (26, 147), but also to the fact that CA-exposed preterm infants would require longer hospitalization and more days of invasive therapies, rendering them more susceptible to LOS.

Besides the meta-regression analysis, other data of the present study underline the modulatory role of GA on the association between CA and LOS. When we performed a subgroup analysis of studies without a significant difference (*p* > 0.05) in GA between the CA-exposed group and the CA-unexposed group, the association between CA and EOS was still strong (odds ratio 3.76), but the association between CA and LOS could not be demonstrated. When we pooled the few studies that corrected for GA, as well as for other potential confounders, the association between CA and EOS was tempered but still positive, and the association between CA and LOS could not be further demonstrated. This effect of the use of adjusted data has been previously described in meta-analyses on the association between CA and bronchopulmonary dysplasia (12, 13), cerebral palsy (22), retinopathy of prematurity (16), patent ductus arteriosus (17), and intraventricular hemorrhage (19). Moreover, the decreased risk of LOS in CA-exposed infants previously reported by Strunk et al. was only observed after correction for GA (25). Adjustment for GA is commonly used in observational studies examining predictors of outcomes in preterm infants (3, 148, 149). However, GA may represent both a risk factor *per se* and a mediator in the causal pathway linking cause of preterm birth (i.e., CA) to outcome (i.e., LOS). As pointed out by Ananth and Schisterman, GA is often mislabeled as a confounder when it may be an intermediate (149). Adjustment for GA in the presence of unmeasured factors that may affect both GA and neonatal outcome, may result in bias (3, 148–151). This bias may even change the direction of estimates, unless all mediator–outcome confounders are taken into account in the analysis, a condition that is unlikely to be achieved (3, 148–152). Therefore, observational studies and meta-analyses of observational studies that analyze the association between conditions like CA or pre-eclampsia and outcome of preterm birth provide valuable information for descriptive or prognostic purposes. However, inferring causal effects is not possible (145, 149).

There is a reasonable biological plausibility for the immunomodulatory role of intrauterine infection/inflammation on the etiopathogenesis of neonatal sepsis. Nevertheless, our comprehension of the mechanisms that link intrauterine infection/inflammation and preterm birth is still incomplete. The maternal lower genital tract is generally considered to be the primary source of bacteria, but other potential sources, such as the oral cavity, need to be considered (7, 8). The microorganisms more frequently cultivated from amniotic fluid in pregnancies complicated by preterm birth are *Ureaplasma urealyticum, Mycoplasma hominis, Fusobacterium nucleatum, Gardnerella vaginalis*, and *Bacteroides* spp. (7, 8). These microorganisms are able to stimulate the intrauterine inflammatory process but have relatively low virulence and rarely induce EOS, which is mainly related to such bacteria as group B streptococcus or *Escherichia coli* (153). In addition, an important proportion of the organisms associated with intra-amniotic infection are uncultivated or difficult-to-cultivate bacteria (7, 8). Nevertheless, as suggested by Strunk et al., even culture-negative, asymptomatic CA that does not result in EOS might lead to a persistent alteration of the neonatal immune system (11). Accordingly, evidence from animal and human studies supports that CA could diminish innate immune responses and thereby increase the susceptibility of preterm infants to LOS (154–157).

Not all the situations of intrauterine infection/inflammation will lead to an inflammatory process reaching the fetus (158). Funisitis is considered the histologic counterpart of the fetal inflammatory response syndrome (9, 158). Our meta-analysis could not demonstrate a stronger association between funisitis and the risk of developing neonatal sepsis (EOS, LOS, or UOS), when compared with CA in the absence of funisitis (Figure 8). This is an argument against the role of the immunomodulation induced by the fetal inflammatory response in the etiopathogenesis of neonatal sepsis. Similarly, we observed in previous meta-analyses that funisitis was not an additional risk factor for developing intraventricular hemorrhage (19), patent ductus arteriosus (17), respiratory distress syndrome (13), or bronchopulmonary dysplasia (13) in preterm infants. In contrast, funisitis significantly increased the risk of retinopathy of prematurity (16). However, all these meta-analyses, as well as the present one, are limited by the small number of studies providing data on funisitis.

Besides the immunomodulatory role of prenatal infection/inflammation, some differences between CA-exposed infants and CA-unexposed infants may also play a role in the association between CA and neonatal sepsis. Our meta-analysis showed a higher rate of exposure to antenatal corticosteroids among CA-exposed infants, but meta-regression could not demonstrate that this affected the association between CA and sepsis. In addition, alterations in infant's microbiome due to mode of delivery, or antibiotic exposure in early life may be related to the increased risk of LOS in CA-exposed infants (159). Concerns that an intrauterine infection is the trigger for prematurity lead to the initiation of empirical antibiotics in the majority of very and extremely preterm infants (160). If the newborn has a true infection, these antibiotics will save his life, but overuse may lead to the development of antibiotic resistance. Moreover, growing evidence shows that prolonged initial empirical antibiotic treatment may be associated with adverse outcomes, such as LOS, necrotizing enterocolitis, bronchopulmonary dysplasia, or death (160, 161).

Our study has several limitations. The studies showed great heterogeneity in their definition of CA, particularly pertaining to criteria used in defining clinical CA. Recent recommendations propose to restrict the term CA to pathologic diagnosis (162). With regard to histological CA, the definition and staging criteria of Redline et al. (163) were the most frequently used but only 11 studies (25, 41, 43, 50, 55, 61, 81, 90, 111, 120, 141) used this classification and only two studies (81, 141) stratified the outcomes according to the grade of histological CA. In addition, few studies had the association between CA and sepsis as their main study objective, while at the same time this may have acted in favor of avoiding publication bias. Furthermore, the generalized definition of EOS, LOS, clinical, and culture-proven sepsis did not allow for analysis of individual pathogen associations. Such an approach was simply not feasible given the overall lack of such specific data across all analyzed studies. Currently, no consensus exists on the definition of neonatal bacterial sepsis unlike pediatric sepsis (164, 165). Pediatric sepsis is defined as a “systemic inflammatory response syndrome in the presence of a suspected or proven infection,” conditions that do not necessarily apply to neonatal sepsis (166). Neonatal sepsis in most cases may present itself with a negative blood culture, and with non-specific clinical symptoms (167). Providing a clear disease definition of neonatal sepsis remains therefore in part challenging due to current limitations of ancillary diagnostics (165, 168).

## Conclusions

Infant's immaturity plays a key role in the morbidity associated with very/extremely preterm birth but the pathological processes causing preterm birth may also influence the outcome. Our data suggest that when infection/inflammation is the trigger of preterm birth, infants are more susceptible to develop sepsis not only immediately after birth, but also during the first weeks of life. The association between CA and EOS seems to be GA-independent, whereas the lower GA of CA-exposed infants modulated the effect size of the association between CA and LOS. CA may initiate the immunomodulatory sequence leading to LOS but also may alter the rate of exposure to other stimuli such as antenatal and post-natal corticosteroids, antenatal and post-natal antibiotics, invasive therapies, lung damage, patent ductus arteriosus, or necrotizing enterocolitis, which render very/extremely preterm infants more vulnerable to sepsis. A meta-analysis of individual patient data would help determine the role of some of these factors in the different outcomes of preterm children exposed to chorioamnionitis.

## Data Availability Statement

The raw data supporting the conclusions of this article will be made available by the authors, without undue reservation, to any qualified researcher.

## Author's Note

This study was presented at the 3rd Congress of Joint European Neonatal Societies (jENS 2019). Abstract available at https://www.nature.com/articles/s41390-019-0521-6.

## Author Contributions

EV-M contributed to the search and inclusion of studies, carried out data collection, carried out statistical analyses, assessed methodological quality, contributed to interpretation of results, helped draft the initial manuscript, and reviewed and revised the manuscript. GL contributed to the search and inclusion of studies, contributed to data collection, assessed methodological quality, contributed to interpretation of results, and drafted the initial manuscript. OR selected studies for inclusion, carried out data collection, and carried out statistical analyses. PD carried out and supervised data collection, contributed to interpretation of results, and reviewed and revised the manuscript. LZ contributed to interpretation of results and reviewed and revised the manuscript. BK contributed to interpretation of results, contributed to drafting the manuscript, and reviewed and revised the manuscript. EV conceptualized and designed the study, carried out the search and selected studies for inclusion, supervised data collection, contributed to statistical analyses and interpretation of results, helped draft the initial manuscript, and reviewed and revised the manuscript. All authors approved the final manuscript as submitted.

## Conflict of Interest

The authors declare that the research was conducted in the absence of any commercial or financial relationships that could be construed as a potential conflict of interest. The handling Editor declared a past co-authorship with one of the authors BK.
