# Supplementary material for: Association of Histological and Clinical Chorioamnionitis With Neonatal Sepsis Among Preterm Infants: A Systematic Review, Meta-Analysis, and Meta-Regression
Source: Front Immunol. 2020 Jun 5;11:972. doi: 10.3389/fimmu.2020.00972 (PMC7289970; doi:10.3389/fimmu.2020.00972)
Supplement: Supplementary file 1 [file Data_Sheet_1.PDF]

Association of Histological and Clinical Chorioamnionitis with Neonatal Sepsis among Preterm Infants: A Systematic Review, Meta-analysis and Meta-regression

Eduardo Villamor-Martinez[1], George A Lubach[1], Owais Mohammed Rahim[1], Pieter Degraeuwe[1], Luc J Zimmermann[1], Boris W Kramer[1], Eduardo Villamor[1]

[1] Department of Pediatrics, Maastricht University Medical Center (MUMC+), School for Oncology and Developmental Biology (GROW), Maastricht, the Netherlands.

Supplementary Material

1.1 Supplementary Tables

Supplementary Table 1. Synoptic table of study characteristics, and Newcastle-Ottawa quality score per study.

First Author, year	Country	Prospective?	Study Design	Perspective	Total infants (centers)	CA Definition	Definition of sepsis	NOS-score			
								Selection	Compar.	Outcome	Total
Adams 1993	USA	No	Case-Control	Sepsis	92 (1)	Unspecified	Proven	4	0	2	6
Ahn 2012	South Korea	Yes	Cohort	CA	257 (1)	Histological	Proven	4	1	3	8
Alam 2014	Pakistan	No	Cohort	Sepsis	428 (1)	Clinical	Proven	4	0	3	7
Alexander 1998	USA	Yes	Cohort	CA	1367 (1)	Clinical	Proven or Clinical	4	2	3	9

First Author, year	Country	Prospective?	Study Design	Perspective	Total infants (centers)	CA Definition	Definition of sepsis	NOS-score			
								Selection	Compar.	Outcome	Total
Alshaikh 2014	Canada	No	Cohort	Sepsis	332 (1)	Unspecified	Proven	3	0	3	6
Anblagan 2016	UK	Yes	Cohort	CA	90 (1)	Histological	Proven or Clinical	4	0	3	7
Arayici 2014	Turkey	No	Cohort	CA	281 (1)	Histological	Proven & Clinical	4	0	3	7
Arora 2015	India	Yes	Cohort	CA-Sepsis	97 (1)	Histological & Clinical	Proven & Clinical	4	0	3	7
Austeng (EXPRESS) 2010	Sweden	Yes	Cohort	CA	497 (7)	Clinical	Unspecified	3	2	3	8
Averbuch 1995	Israel	Yes	Cohort	CA	90 (1)	Histological	Proven	4	0	3	7
Aziz 2009	USA	No	Cohort	CA	1153 (1)	Clinical	Proven	4	2	3	9
Ballard 2016	USA	Yes	Cohort	CA	1687 (1)	Clinical	Proven	4	1	3	8
Barrera-Reyes 2011	Mexico	Yes	Cohort	CA	104 (1)	Clinical	Unspecified	4	0	3	7
Been 2010	Netherlands	Yes	Cohort	CA	301 (1)	Histological	Proven	4	2	3	9
Bordigato 2011	Italy	Yes	Cohort	CA	29 (1)	Histological	Proven	4	0	3	7
Botet 2011	Spain	Yes	Case-Control	CA	328 (1)	Clinical	Proven & Clinical	3	0	3	6
Bry 2015	Sweden	Unknown	Cohort	CA	24 (1)	Histological	Unspecified	4	0	3	7
Buhimschi 2007	USA	Yes	Cohort	CA-Sepsis	123 (1)	Histological	Proven & Clinical	4	0	3	7
Buhimschi 2009	USA	Yes	Cohort	CA	132 (1)	Histological	Proven	4	0	3	7
Burgner, D.P. 2017	Australia	No	Cohort	CA	1218 (1)	Histological	Unspecified	4	0	3	7
Chau 2012	Canada	Yes	Cohort	Sepsis	117 (1)	Histological	Proven	2	0	3	5
Choi 2008	South Korea	Yes	Cohort	CA	62 (2)	Histological	Proven	4	0	3	7
Churgay 1994	USA	No	Cohort	CA	81 (1)	Clinical	Clinical	3	0	3	6
De Felice 2005	Italy	Yes	Cohort	CA	115 (2)	Histological	Unspecified	4	0	3	7

First Author, year	Country	Prospective?	Study Design	Perspective	Total infants (centers)	CA Definition	Definition of sepsis	NOS-score			
								Selection	Compar.	Outcome	Total
Dempsey 2005	Canada	Yes	Cohort	CA	330 (1)	Histological	Proven	4	0	3	7
Dessardo 2014	Croatia	Yes	Cohort	CA	262 (1)	Histological	Clinical	4	0	3	7
Dexter 1999	USA	Unknown	Cohort	CA	330 (1)	Clinical	Proven	4	0	3	7
Dexter 2000	USA	Unknown	Cohort	CA	287 (1)	Histological	Unspecified	4	0	3	7
Dollner 2002	Norway	Yes	Cohort	CA	221 (1)	Histological	Proven & Clinical	3	0	3	6
Dulay 2008	USA	Yes	Cohort	Sepsis	68 (1)	Clinical	Proven	3	0	3	6
Dutta 2010	India	Yes	Cohort	Sepsis	601 (1)	Clinical	Proven	4	2	3	9
Ecevit 2014	Spanje	No	Cohort	CA	19 (1)	Histological	Clinical	4	0	3	7
Elimian 2000	USA	No	Cohort	CA	1260 (1)	Histological	Proven	3	2	3	8
Erdemir 2013	Turkey	Yes	Cohort	CA	56 (19	Histological	Clinical	3	0	3	6
Figueroa-Damian 1999	Mexico	Yes	Cohort	Sepsis	92 (1)	Clinical	Clinical	3	0	3	6
Gagliardi 2014	Italy	Yes	Cohort	CA	3605 (82)	Clinical	Proven	3	2	3	8
Garcia-Munoz Rodrigo 2014	Spain	No	Cohort	CA	8329 (53)	Clinical	Proven	4	2	3	9
Gonzalez-Luis 2002	Spain	No	Case-Control	CA	135 (1)	Clinical	Proven & Clinical	4	0	3	7
Guzick 1985	USA	Yes	Cohort	CA	2774 (1)	Histological	Unspecified	4	0	3	7
Gyamfi-Bannerman 2014	USA	Yes	Cohort	Sepsis	1640 (20)	Unspecified	Proven	3	2	3	8
Hendson 2011	UK	Yes	Cohort	CA	622 (1)	Histological	Proven	4	0	3	7
Hitti 2001	USA/Sweden	Yes	Cohort	CA	139 (3)	Microbiological	Proven	4	0	3	7
Howman 2012	Australia	Yes	Cohort	CA	325 (1)	Histological	Clinical	4	0	3	7
Janota 2001	Czech Republic	Yes	Cohort	CA-Sepsis	37 (1)	Histological	Proven or Clinical	4	0	3	7
Jeon 2014	South	Yes	Case-	Sepsis	126 (1)	Clinical	Proven &	3	0	3	6

First Author, year	Country	Prospective?	Study Design	Perspective	Total infants (centers)	CA Definition	Definition of sepsis	NOS-score			
								Selection	Compar.	Outcome	Total
	Korea		Control				Clinical				
Jones 2013	Brazil	Yes	Cohort	CA	95 (1)	Histological	Clinical	4	0	3	7
Kacerovsky 2014	Czech Republic	Yes	Cohort	CA	99 (1)	Histological	Proven & Clinical	3	0	3	6
Kim 2015	South Korea	No	Cohort	CA	258 (1)	Histological	Proven	4	0	3	7
Kirchner 2007	Austria	No	Cohort	CA	44 (1)	Microbiological	Proven	4	0	3	7
Klinger 2009	Israel	Yes	Cohort	Sepsis	15838 (28)	Clinical	Proven	4	0	3	7
Lahra 2009	Australia	Yes	Cohort	CA	761 (1)	Histological	Proven	4	0	3	7
Lau 2005	Canada	Yes	Cohort	CA	1296 (1)	Histological	Proven	4	0	3	7
Lee SY 2012	South Korea	No	Cohort	Sepsis	306 (1)	Histological	Proven	4	0	3	7
Lee SYR & Leung 2012	China	No	Cohort	CA-Sepsis	258 (1)	Histological	Proven	4	0	3	7
Lee Y 2015	South Korea	No	Cohort	CA	339 (1)	Histological	Proven	4	2	3	9
Liu 2014	China	Yes	Cohort	CA	216 (1)	Histological	Clinical	4	0	3	7
Lorthe, R.M. 2017	France	Yes	Cohort	Sepsis	664 (546)	Clinical	Proven	2	1	3	6
Maziad 2010	USA	No	Case-Control	Sepsis	399 (1)	Clinical	Proven	4	0	2	6
Mehta 2006	USA	No	Cohort	Sepsis	164 (1)	Histological	Proven	4	0	3	7
Mikhael 2014	USA	No	Cohort	Sepsis	1584 (1)	Clinical	Clinical	3	0	3	6
Miyazaki 2016	Japan	No	Cohort	CA	4077 (?)	Histological	Proven	3	2	3	8
Mu 2008	Taiwan	Yes	Cohort	CA	119 (1)	Histological	Proven	4	1	3	8
Mwanyumba 2003	Kenya	Yes	Cohort	CA	349 (1)	Histological	Clinical	4	0	3	7
Nasef 2013	Canada	No	Cohort	CA	241 (1)	Histological	Unspecified	4	2	3	9

First Author, year	Country	Prospective?	Study Design	Perspective	Total infants (centers)	CA Definition	Definition of sepsis	NOS-score			
								Selection	Compar.	Outcome	Total
Ofman 2016	USA	No	Cohort	CA-Sepsis	2192 (1)	Clinical	Proven & Clinical	4	2	3	9
Ogunyemi 2003	USA	No	Cohort	CA	266 (1)	Histological	Proven	4	2	3	9
Oh 2015	South Korea	Yes	Cohort	CA	91 (1)	Histological	Unspecified	4	0	3	7
Ohyama 2002	Japan	No	Cohort	CA	143 (1)	Histological	Proven & Clinical	4	0	3	7
O'shea 1998	USA	Yes	Case-Control	CA	131 (1)	Histological & Clinical	Unspecified	4	0	3	7
Ozkan 2014	Turkey	Yes	Cohort	Sepsis	151 (1)	Unspecified	Proven	3	0	3	6
Pappas 2013	USA	No	Cohort	CA	2390 (1)	Histological	Unspecified	4	0	3	7
Park 2004	South Korea	Yes	Cohort	Sepsis	59 (1)	Histological & Clinical	Proven & Clinical	4	0	3	7
Park, J.W. 2017	South Korea	No	Cohort	CA	153 (1)	Microbiological	Proven & Clinical	4	0	3	7
Plakkal 2013	Canada	No	Cohort	CA	529 (1)	Funisitis	Proven	4	0	3	7
Popowski 2011	France	Yes	Cohort	Sepsis	398 (2)	Clinical	Proven	4	0	3	7
Prendergast 2011	UK	No	Cohort	CA	120 (1)	Histological	Unspecified	4	0	3	7
Pristauz 2009	Austria	No	Cohort	CA	25 (1)	Histological	Clinical	4	0	3	7
Puri, K. 2016	USA	Yes	Case-Control	CA	106 (1)	Histological	Proven	4	0	3	7
Rocha 2006	Portugal	No	Case-Control	CA	452 (1)	Histological	Proven	4	0	3	7
Rodriguez-Trujillo 2016	Spain	Yes	Cohort	CA	237 (1)	Microbiological	Clinical	3	0	3	6
Ronnestad 2005	Norway	Yes	Cohort	Sepsis	461 (21)	Clinical	Proven	3	1	3	7
Salem 2006	Israel	No	Case-Control	Sepsis	786 (1)	Clinical	Unspecified	3	0	3	6
Sato 2011	Japan	No	Cohort	CA	301 (1)	Histological	Proven	4	0	3	7
Schlapbach 2010	Switzerland	Yes	Case-	CA	99 (1)	Histological &	Proven	3	0	3	6

First Author, year	Country	Prospective?	Study Design	Perspective	Total infants (centers)	CA Definition	Definition of sepsis	NOS-score Selection	Compar.	Outcome	Total
			Control			Clinical					
Schuchat 2000	USA	No	Case-Control	Sepsis	302 (1)	Clinical	Proven	4	0	3	7
Shah 2015	Canada	No	Cohort	Sepsis	7508 (29)	Clinical	Proven	4	0	3	7
Smit 2015	Netherlands	Yes	Cohort	CA	548 (1)	Histological	Proven & Clinical	4	0	3	7
Smulian 1999	USA	No	Cohort	CA	139 (1)	Histological	Clinical	4	0	3	7
Soraisham 2009	Canada	Yes	Cohort	CA	3093 (24)	Clinical	Proven	4	2	3	9
Soraisham 2013	Canada	No	Cohort	CA	384 (1)	Histological	Proven	4	0	3	7
Stepan 2016	Czech Republic	Yes	Cohort	CA	71 (1)	Histological	Proven & Clinical	3	0	3	6
Stimac 2014	Croatia	No	Cohort	CA	395 (1)	Histological	Proven & Clinical	4	0	3	7
Stranak 2016	Czech Republic	No	Cohort	CA	91 (1)	Histological & Clinical	Proven or Clinical	4	0	3	7
Strunk 2012	Australia	No	Cohort	CA-Sepsis	838 (1)	Histological	Proven & Clinical	4	2	3	9
Sung 2017	South Korea	No	Cohort	CA	1468 (1)	Clinical	Proven	4	0	3	7
Trevisanuto 2013	Italy	No	Case-Control	CA	142 (1)	Histological	Unspecified	3	0	3	6
Tsai 2012	China	No	Case-Control	Sepsis	46 (1)	Unspecified	Proven or Clinical	3	0	2	5
Tsiartas 2013	Czech Republic	3	Cohort	CA	231 (1)	Histological	Proven or Clinical	4	0	3	7
Tudela 2012	USA	No	Cohort	Sepsis	145065 (1)	Unspecified	Proven	3	0	3	6
Van Marter 2002	USA	No	Case-Control	CA	256 (3)	Histological	Clinical	4	0	3	7
Van Vliet 2012	Netherlands	Yes	Cohort	CA	71 (1)	Histological	Proven	4	2	3	9

First Author, year	Country	Prospective?	Study Design	Perspective	Total infants (centers)	CA Definition	Definition of sepsis	NOS-score			
								Selection	Compar.	Outcome	Total
Vander Haar 2016	USA	No	Cohort	CA	1573 (20)	Clinical	Proven	3	0	2	5
Vinnars 2015	Sweden	Yes	Cohort	CA	167 (1)	Histological	Proven	4	0	3	7
Watterberg 1999	USA	Yes	RCT	CA	62 (1)	Histological	Proven	2	0	3	5
Wynn 2013	USA	No	Cohort	Sepsis	7513 (30)	Histological	Proven	3	0	3	6
Xie 2015	China	No	Cohort	CA	371 (1)	Histological	Unspecified	3	0	3	6
Yancey 1996	USA	Yes	Cohort	Sepsis	823 (1)	Clinical	Proven	4	1	3	8
Yoon 1995	South Korea	Yes	Cohort	CA	50 (1)	Histological	Unspecified	3	0	3	6
**Total of 107 Studies**					**Total Infants = 387,321**						

Perspective refers to whether the article primarily studied chorioamnionitis, neonatal sepsis or specifically the association between chorioamnionitis and neonatal sepsis (denoted as CA-Sepsis).
CA: chorioamnionitis; Compar.: comparison; NOS: Newcastle-Ottawa Scale; proven: refers to culture-proven sepsis.

List of included studies

Abu-Maziad, A., K. Schaa, E. F. Bell, J. M. Dagle, M. Cooper, M. L. Marazita and J. C. Murray (2010). "Role of polymorphic variants as genetic modulators of infection in neonatal sepsis." Pediatr Res **68**(4): 323-329.

Adams, W. G., J. S. Kinney, A. Schuchat, C. L. Collier, C. J. Papasian, H. W. Kilbride, F. X. Riedo and C. V. Broome (1993). "Outbreak of early onset group B streptococcal sepsis." Pediatr Infect Dis J **12**(7): 565-570.

Ahn, H. M., E. A. Park, S. J. Cho, Y. J. Kim and H. S. Park (2012). "The association of histological chorioamnionitis and antenatal steroids on neonatal outcome in preterm infants born at less than thirty-four weeks' gestation." Neonatology **102**(4): 259-264.

Alam, M. M., A. F. Saleem, A. S. Shaikh, O. Munir and M. Qadir (2014). "Neonatal sepsis following prolonged rupture of membranes in a tertiary care hospital in Karachi, Pakistan." J Infect Dev Ctries **8**(1): 67-73.

Alexander, J. M., L. C. Gilstrap, S. M. Cox, D. M. McIntire and K. J. Leveno (1998). "Clinical chorioamnionitis and the prognosis for very low birth weight infants." Obstet Gynecol **91**(5 Pt 1): 725-729.

Alfiero Bordigato, M., D. Piva, I. M. Di Gangi, G. Giordano, L. Chiandetti and M. Filippone (2011). "Asymmetric dimethylarginine in ELBW newborns exposed to chorioamnionitis." Early Hum Dev **87**(2): 143-145.

Alshaikh, B., W. Yee, A. Lodha, E. Henderson, K. Yusuf and R. Sauve (2014). "Coagulase-negative staphylococcus sepsis in preterm infants and long-term neurodevelopmental outcome." J Perinatol **34**(2): 125-129.

Anblagan, D., R. Pataky, M. J. Evans, E. J. Telford, A. Serag, S. Sparrow, C. Piyasena, S. I. Semple, A. G. Wilkinson, M. E. Bastin and J. P. Boardman (2016). "Association between preterm brain injury and exposure to chorioamnionitis during fetal life." Sci Rep **6**: 37932.

Arayici, S., G. Kadioglu Simsek, M. Y. Oncel, Z. Eras, F. E. Canpolat, S. S. Oguz, N. Uras, S. Zergeroglu and U. Dilmen (2014). "The effect of histological chorioamnionitis on the short-term outcome of preterm infants </=32 weeks: a single-center study." J Matern Fetal Neonatal Med **27**(11): 1129-1133.

Arora, P., R. Bagga, J. Kalra, P. Kumar, S. Radhika and V. Gautam (2015). "Mean gestation at delivery and histological chorioamnionitis correlates with early-onset neonatal sepsis following expectant management in pPROM." J Obstet Gynaecol **35**(3): 235-240.

Averbuch, B., M. Mazor, I. Shoham-Vardi, W. Chaim, H. Vardi, S. Horowitz and M. Shuster (1995). "Intra-uterine infection in women with preterm premature rupture of membranes: maternal and neonatal characteristics." Eur J Obstet Gynecol Reprod Biol **62**(1): 25-29.

Aziz, N., Y. W. Cheng and A. B. Caughey (2009). "Neonatal outcomes in the setting of preterm premature rupture of membranes complicated by chorioamnionitis." J Matern Fetal Neonatal Med **22**(9): 780-784.

Ballard, A. R., L. H. Mallett, J. E. Pruszynski and J. B. Cantey (2016). "Chorioamnionitis and subsequent bronchopulmonary dysplasia in very-low-birth weight infants: a 25-year cohort." J Perinatol **36**(12): 1045-1048.

Barrera-Reyes, R. H., H. Ruiz-Macias and E. Segura-Cervantes (2011). "[Neurodevelopment at one year of age [corrected] in preterm newborns with history of maternal chorioamnionitis]." Ginecol Obstet Mex **79**(1): 31-37.

Been, J. V., I. G. Rours, R. F. Kornelisse, V. Lima Passos, B. W. Kramer, T. A. Schneider, R. R. de Krijger and L. J. Zimmermann (2009). "Histologic chorioamnionitis, fetal involvement, and antenatal steroids: effects on neonatal outcome in preterm infants." Am J Obstet Gynecol **201**(6): 587 e581-588.

Botet, F., J. Figueras, X. Carbonell-Estrany, G. Arca and G. The Castrillo Study (2010). "Effect of maternal clinical chorioamnionitis on neonatal morbidity in very-low birthweight infants: a case-control study." J Perinat Med **38**(3): 269-273.

Braun, D., P. Bromberger, N. J. Ho and D. Getahun (2016). "Low Rate of Perinatal Sepsis in Term Infants of Mothers with Chorioamnionitis." Am J Perinatol **33**(2): 143-150.

Bry, K. J., B. Jacobsson, S. Nilsson and K. Bry (2015). "Gastric fluid cytokines are associated with chorioamnionitis and white blood cell counts in preterm infants." Acta Paediatr **104**(6): 575-580.

Buhimschi, C. S., I. A. Buhimschi, S. Abdel-Razeq, V. A. Rosenberg, S. F. Thung, G. Zhao, E. Wang and V. Bhandari (2007). "Proteomic biomarkers of intra-amniotic inflammation: relationship with funisitis and early-onset sepsis in the premature neonate." Pediatr Res **61**(3): 318-324.

Buhimschi, C. S., A. T. Dulay, S. Abdel-Razeq, G. Zhao, S. Lee, E. J. Hodgson, V. Bhandari and I. A. Buhimschi (2009). "Fetal inflammatory response in women with proteomic biomarkers characteristic of intra-amniotic inflammation and preterm birth." BJOG **116**(2): 257-267.

Burgner, D. P., D. Doherty, J. Humphreys, A. Currie, K. Simmer, A. Charles and T. Strunk (2017). "Maternal Chorioamnionitis and Postneonatal Respiratory Tract Infection in Ex-Preterm Infants." J Pediatr **184**: 62-67 e62.
Chau, V., R. Brant, K. J. Poskitt, E. W. Tam, A. Synnes and S. P. Miller (2012). "Postnatal infection is associated with widespread abnormalities of brain development in premature newborns." Pediatr Res **71**(3): 274-279.
Choi, C. W., B. I. Kim, K. E. Joung, J. A. Lee, Y. K. Lee, E. K. Kim, H. S. Kim, J. D. Park and J. H. Choi (2008). "Decreased expression of transforming growth factor-beta1 in bronchoalveolar lavage cells of preterm infants with maternal chorioamnionitis." J Korean Med Sci **23**(4): 609-615.
Churgay, C. A., M. A. Smith and B. Blok (1994). "Maternal fever during labor--what does it mean?" J Am Board Fam Pract **7**(1): 14-24.
De Felice, C., P. Toti, S. Parrini, A. Del Vecchio, F. Bagnoli, G. Latini and R. J. Kopotic (2005). "Histologic chorioamnionitis and severity of illness in very low birth weight newborns." Pediatr Crit Care Med **6**(3): 298-302.
Dempsey, E., M. F. Chen, T. Kokottis, D. Vallerand and R. Usher (2005). "Outcome of neonates less than 30 weeks gestation with histologic chorioamnionitis." Am J Perinatol **22**(3): 155-159.
Dessardo, N. S., S. Dessardo, E. Mustac, S. Banac, O. Petrovic and B. Peter (2014). "Chronic lung disease of prematurity and early childhood wheezing: is foetal inflammatory response syndrome to blame?" Early Hum Dev **90**(9): 493-499.
Dexter, S. C., M. P. Malee, H. Pinar, J. W. Hogan, M. W. Carpenter and B. R. Vohr (1999). "Influence of chorioamnionitis on developmental outcome in very low birth weight infants." Obstet Gynecol **94**(2): 267-273.
Dexter, S. C., H. Pinar, M. P. Malee, J. Hogan, M. W. Carpenter and B. R. Vohr (2000). "Outcome of very low birth weight infants with histopathologic chorioamnionitis." Obstet Gynecol **96**(2): 172-177.
Dollner, H., L. Vatten, J. Halgunset, S. Rahimipoor and R. Austgulen (2002). "Histologic chorioamnionitis and umbilical serum levels of pro-inflammatory cytokines and cytokine inhibitors." BJOG **109**(5): 534-539.
Dulay, A. T., I. A. Buhimschi, G. Zhao, G. Luo, S. Abdel-Razeq, M. Cackovic, V. A. Rosenberg, C. M. Pettker, S. F. Thung, M. O. Bahtiyar, V. Bhandari and C. S. Buhimschi (2008). "Nucleated red blood cells are a direct response to mediators of inflammation in newborns with early-onset neonatal sepsis." Am J Obstet Gynecol **198**(4): 426 e421-429.
Dutta, S., R. Reddy, S. Sheikh, J. Kalra, P. Ray and A. Narang (2010). "Intrapartum antibiotics and risk factors for early onset sepsis." Arch Dis Child Fetal Neonatal Ed **95**(2): F99-103.
Ecevit, A., D. Anuk-Ince, E. Yapakci, S. Kupana-Ayva, A. Kurt, F. F. Yanik and A. Tarcan (2014). "Association of respiratory distress syndrome and perinatal hypoxia with histologic chorioamnionitis in preterm infants." Turk J Pediatr **56**(1): 56-61.
Elimian, A., U. Verma, D. Beneck, R. Cipriano, P. Visintainer and N. Tejani (2000). "Histologic chorioamnionitis, antenatal steroids, and perinatal outcomes." Obstet Gynecol **96**(3): 333-336.
Erdemir, G., N. Kultursay, S. Calkavur, O. Zekioglu, O. A. Koroglu, B. Cakmak, M. Yalaz, M. Akisu and S. Sagol (2013). "Histological chorioamnionitis: effects on premature delivery and neonatal prognosis." Pediatr Neonatol **54**(4): 267-274.
Figueroa-Damian, R., J. L. Arredondo-Garcia and J. Mancilla-Ramirez (1999). "Amniotic fluid interleukin-6 and the risk of early-onset sepsis among preterm infants." Arch Med Res **30**(3): 198-202.
Gagliardi, L., F. Rusconi, R. Bellu, R. Zanini and N. Italian Neonatal (2014). "Association of maternal hypertension and chorioamnionitis with preterm outcomes." Pediatrics **134**(1): e154-161.
Garcia-Munoz Rodrigo, F., G. Galan Henriquez, J. Figueras Aloy and A. Garcia-Alix Perez (2014). "Outcomes of very-low-birth-weight infants exposed to maternal clinical chorioamnionitis: a multicentre study." Neonatology **106**(3): 229-234.
Gonzalez-Luis, G., I. Jordan Garcia, J. Rodriguez-Miguelez, F. Botet Mussons and J. Figueras Aloy (2002). "[Neonatal morbidity and mortality in very low birth weight infants according to exposure to chorioamnionitis]." An Esp Pediatr **56**(6): 551-555.
Group, E. (2010). "Incidence of and risk factors for neonatal morbidity after active perinatal care: extremely preterm infants study in Sweden (EXPRESS)." Acta Paediatr **99**(7): 978-992.
Guzick, D. S. and K. Winn (1985). "The association of chorioamnionitis with preterm delivery." Obstet Gynecol **65**(1): 11-16.
Gyamfi-Bannerman, C. and M. Son (2014). "Preterm premature rupture of membranes and the rate of neonatal sepsis after two courses of antenatal corticosteroids." Obstet Gynecol **124**(5): 999-1003.
Hendson, L., L. Russell, C. M. Robertson, Y. Liang, Y. Chen, A. Abdalla and T. Lacaze-Masmonteil (2011). "Neonatal and neurodevelopmental outcomes of very low birth weight infants with histologic chorioamnionitis." J Pediatr **158**(3): 397-402.
Hitti, J., P. Tarczy-Hornoch, J. Murphy, S. L. Hillier, J. Aura and D. A. Eschenbach (2001). "Amniotic fluid infection, cytokines, and adverse outcome among infants at 34 weeks' gestation or less." Obstet Gynecol **98**(6): 1080-1088.

Howman, R. A., A. K. Charles, A. Jacques, D. A. Doherty, K. Simmer, T. Strunk, P. C. Richmond, C. H. Cole and D. P. Burgner (2012). "Inflammatory and haematological markers in the maternal, umbilical cord and infant circulation in histological chorioamnionitis." PLoS One **7**(12): e51836.
Janota, J., Z. Stranak, S. Belohlavkova, K. Mudra and J. Simak (2001). "Postnatal increase of procalcitonin in premature newborns is enhanced by chorioamnionitis and neonatal sepsis." Eur J Clin Invest **31**(11): 978-983.
Jeon, J. H., R. Namgung, M. S. Park, K. I. Park and C. Lee (2014). "Positive maternal C-reactive protein predicts neonatal sepsis." Yonsei Med J **55**(1): 113-117.
Jones, M. H., A. L. Corso, R. S. Tepper, M. I. Edelweiss, L. Friedrich, P. M. Pitrez and R. T. Stein (2013). "Chorioamnionitis and subsequent lung function in preterm infants." PLoS One **8**(12): e81193.
Kacerovsky, M., I. Musilova, C. Andrys, H. Hornychova, L. Pliskova, M. Kostal and B. Jacobsson (2014). "Prelabor rupture of membranes between 34 and 37 weeks: the intraamniotic inflammatory response and neonatal outcomes." Am J Obstet Gynecol **210**(4): 325 e321-325 e310.
Kim, S. Y., C. W. Choi, E. Jung, J. Lee, J. A. Lee, H. Kim, E. K. Kim, H. S. Kim, B. I. Kim and J. H. Choi (2015). "Neonatal Morbidities Associated with Histologic Chorioamnionitis Defined Based on the Site and Extent of Inflammation in Very Low Birth Weight Infants." J Korean Med Sci **30**(10): 1476-1482.
Kirchner, L., H. Helmer, G. Heinze, M. Wald, M. Brunbauer, M. Weninger and D. Zaknun (2007). "Amnionitis with Ureaplasma urealyticum or other microbes leads to increased morbidity and prolonged hospitalization in very low birth weight infants." Eur J Obstet Gynecol Reprod Biol **134**(1): 44-50.
Klinger, G., I. Levy, L. Sirota, V. Boyko, B. Reichman, L. Lerner-Geva and N. Israel Neonatal (2009). "Epidemiology and risk factors for early onset sepsis among very-low-birthweight infants." Am J Obstet Gynecol **201**(1): 38 e31-36.
Lahra, M. M., P. J. Beeby and H. E. Jeffery (2009). "Intrauterine inflammation, neonatal sepsis, and chronic lung disease: a 13-year hospital cohort study." Pediatrics **123**(5): 1314-1319.
Lau, J., F. Magee, Z. Qiu, J. Hoube, P. Von Dadelszen and S. K. Lee (2005). "Chorioamnionitis with a fetal inflammatory response is associated with higher neonatal mortality, morbidity, and resource use than chorioamnionitis displaying a maternal inflammatory response only." Am J Obstet Gynecol **193**(3 Pt 1): 708-713.
Lee, S. Y. and C. W. Leung (2012). "Histological chorioamnionitis - implication for bacterial colonization, laboratory markers of infection, and early onset sepsis in very-low-birth-weight neonates." J Matern Fetal Neonatal Med **25**(4): 364-368.
Lee, S. Y., K. H. Park, E. H. Jeong, K. J. Oh, A. Ryu and K. U. Park (2012). "Relationship between maternal serum C-reactive protein, funisitis and early-onset neonatal sepsis." J Korean Med Sci **27**(6): 674-680.
Lee, Y., H.-J. Kim, S.-J. Choi, S.-y. Oh, J.-S. Kim, C.-R. Roh and J.-H. Kim (2015). "Is there a stepwise increase in neonatal morbidities according to histological stage (or grade) of acute chorioamnionitis and funisitis?: effect of gestational age at delivery." Journal of perinatal medicine **43**(2): 259-267.
Liu, Z., Z. Tang, J. Li and Y. Yang (2014). "Effects of placental inflammation on neonatal outcome in preterm infants." Pediatr Neonatol **55**(1): 35-40.
Lorthe, E., P. Y. Ancel, H. Torchin, M. Kaminski, B. Langer, D. Subtil, L. Sentilhes, C. Arnaud, B. Carbonne, T. Debillon, P. Delorme, C. D'Ercole, M. Dreyfus, C. Lebeaux, J. E. Galimard, C. Vayssiere, N. Winer, L. F. L'Helias, F. Goffinet and G. Kayem (2017). "Impact of Latency Duration on the Prognosis of Preterm Infants after Preterm Premature Rupture of Membranes at 24 to 32 Weeks' Gestation: A National Population-Based Cohort Study." J Pediatr **182**: 47-52 e42.
Mehta, R., S. Nanjundaswamy, S. Shen-Schwarz and A. Petrova (2006). "Neonatal morbidity and placental pathology." Indian J Pediatr **73**(1): 25-28.
Mikhael, M., L. S. Brown and C. R. Rosenfeld (2014). "Serial neutrophil values facilitate predicting the absence of neonatal early-onset sepsis." J Pediatr **164**(3): 522-528 e521-523.
Miyazaki, K., M. Furuhashi, K. Ishikawa, K. Tamakoshi, K. Hayashi, A. Kai, H. Ishikawa, N. Murabayashi, T. Ikeda, Y. Kono, S. Kusuda and M. Fujimura (2016). "Impact of chorioamnionitis on short- and long-term outcomes in very low birth weight preterm infants: the Neonatal Research Network Japan." J Matern Fetal Neonatal Med **29**(2): 331-337.
Mu, S. C., C. H. Lin, Y. L. Chen, H. J. Ma, J. S. Lee, M. I. Lin, C. C. Lee, T. J. Chen, G. M. Jow and T. C. Sung (2008). "Impact on neonatal outcome and anthropometric growth in very low birth weight infants with histological chorioamnionitis." J Formos Med Assoc **107**(4): 304-310.
Mwanyumba, F., I. Inion, P. Gaillard, K. Mandaliya, M. Praet and M. Temmerman (2003). "Placental inflammation and perinatal outcome." Eur J Obstet Gynecol Reprod Biol **108**(2): 164-170.
Nasef, N., A. E. Shabaan, P. Schurr, D. Iaboni, J. Choudhury, P. Church and M. S. Dunn (2013). "Effect of clinical and histological chorioamnionitis on the outcome of preterm infants." Am J Perinatol **30**(1): 59-68.

O'Shea, T. M., K. L. Klinepeter, P. J. Meis and R. G. Dillard (1998). "Intrauterine infection and the risk of cerebral palsy in very low-birthweight infants." Paediatr Perinat Epidemiol **12**(1): 72-83.
Ofman, G., N. Vasco and J. B. Cantey (2016). "Risk of Early-Onset Sepsis following Preterm, Prolonged Rupture of Membranes with or without Chorioamnionitis." Am J Perinatol **33**(4): 339-342.
Ogunyemi, D., M. Murillo, U. Jackson, N. Hunter and B. Alperson (2003). "The relationship between placental histopathology findings and perinatal outcome in preterm infants." J Matern Fetal Neonatal Med **13**(2): 102-109.
Oh, S.-H., J.-j. Kim, H.-j. Do, B. S. Lee, K.-S. Kim and E. A.-R. Kim (2015). "Preliminary Study on Neurodevelopmental Outcome and Placental Pathology among Extremely Low Birth Weight Infants." Korean J Perinatol **26**(1): 67-77.
Ohyama, M., Y. Itani, M. Yamanaka, A. Goto, K. Kato, R. Ijiri and Y. Tanaka (2002). "Re-evaluation of chorioamnionitis and funisitis with a special reference to subacute chorioamnionitis." Hum Pathol **33**(2): 183-190.
Ozkan, H., M. Cetinkaya, N. Koksal, S. Celebi and M. Hacimustafaoglu (2014). "Culture-proven neonatal sepsis in preterm infants in a neonatal intensive care unit over a 7 year period: coagulase-negative Staphylococcus as the predominant pathogen." Pediatr Int **56**(1): 60-66.
Pappas, A., D. E. Kendrick, S. Shankaran, B. J. Stoll, E. F. Bell, A. R. Laptook, M. C. Walsh, A. Das, E. C. Hale, N. S. Newman, R. D. Higgins, H. Eunice Kennedy Shriver National Institute of Child and N. Human Development Neonatal Research (2014). "Chorioamnionitis and early childhood outcomes among extremely low-gestational-age neonates." JAMA Pediatr **168**(2): 137-147.
Park, J. W., K. H. Park and E. Y. Jung (2017). "Clinical significance of histologic chorioamnionitis with a negative amniotic fluid culture in patients with preterm labor and premature membrane rupture." PLoS One **12**(3): e0173312.
Park, K. H., B. H. Yoon, S. S. Shim, J. K. Jun and H. C. Syn (2004). "Amniotic fluid tumor necrosis factor-alpha is a marker for the prediction of early-onset neonatal sepsis in preterm labor." Gynecol Obstet Invest **58**(2): 84-90.
Plakkal, N., A. S. Soraisham, C. Trevenen, E. A. Freiheit and R. Sauve (2013). "Histological chorioamnionitis and bronchopulmonary dysplasia: a retrospective cohort study." J Perinatol **33**(6): 441-445.
Popowski, T., F. Goffinet, F. Maillard, T. Schmitz, S. Leroy and G. Kayem (2011). "Maternal markers for detecting early-onset neonatal infection and chorioamnionitis in cases of premature rupture of membranes at or after 34 weeks of gestation: a two-center prospective study." BMC Pregnancy Childbirth **11**: 26.
Prendergast, M., C. May, S. Broughton, E. Pollina, A. D. Milner, G. F. Rafferty and A. Greenough (2011). "Chorioamnionitis, lung function and bronchopulmonary dysplasia in prematurely born infants." Arch Dis Child Fetal Neonatal Ed **96**(4): F270-274.
Pristauz, G., A. A. Bader, G. Schwantzer, J. Kutschera and U. Lang (2009). "Assessment of risk factors for survival of neonates born after second-trimester PPROM." Early Hum Dev **85**(3): 177-180.
Puri, K., D. H. Taft, N. Ambalavanan, K. R. Schibler, A. L. Morrow and S. G. Kallapur (2016). "Association of Chorioamnionitis with Aberrant Neonatal Gut Colonization and Adverse Clinical Outcomes." PLoS One **11**(9): e0162734.
Rocha, G., E. Proenca, C. Quintas, T. Rodrigues and H. Guimaraes (2006). "[Chorioamnionitis and neonatal morbidity]." Acta Med Port **19**(3): 207-212.
Rodriguez-Trujillo, A., T. Cobo, I. Vives, J. Bosch, M. Kacerovsky, D. E. Posadas, M. A. Angeles, E. Gratacos, B. Jacobsson and M. Palacio (2016). "Gestational age is more important for short-term neonatal outcome than microbial invasion of the amniotic cavity or intra-amniotic inflammation in preterm prelabor rupture of membranes." Acta Obstet Gynecol Scand **95**(8): 926-933.
Ronnestad, A., T. G. Abrahamsen, S. Medbo, H. Reigstad, K. Lossius, P. I. Kaaresen, I. E. Engelund, L. M. Irgens and T. Markestad (2005). "Septicemia in the first week of life in a Norwegian national cohort of extremely premature infants." Pediatrics **115**(3): e262-268.
Salem, S. Y., E. Sheiner, E. Zmora, H. Vardi, I. Shoham-Vardi and M. Mazor (2006). "Risk factors for early neonatal sepsis." Arch Gynecol Obstet **274**(4): 198-202.
Sato, M., S. Nishimaki, S. Yokota, K. Seki, H. Horiguchi, H. An, F. Ishida, S. Fujita, K. Ao and H. Yatake (2011). "Severity of chorioamnionitis and neonatal outcome." J Obstet Gynaecol Res **37**(10): 1313-1319.
Schlapbach, L. J., J. Ersch, M. Adams, V. Bernet, H. U. Bucher and B. Latal (2010). "Impact of chorioamnionitis and preeclampsia on neurodevelopmental outcome in preterm infants below 32 weeks gestational age." Acta Paediatr **99**(10): 1504-1509.
Schuchat, A., S. S. Zywicki, M. J. Dinsmoor, B. Mercer, J. Romaguera, M. J. O'Sullivan, D. Patel, M. T. Peters, B. Stoll and O. S. Levine (2000). "Risk factors and opportunities for prevention of early-onset neonatal sepsis: a multicenter case-control study." Pediatrics **105**(1 Pt 1): 21-26.
Shah, J., A. L. Jefferies, E. W. Yoon, S. K. Lee, P. S. Shah and N. Canadian Neonatal (2015). "Risk Factors and Outcomes of Late-Onset Bacterial Sepsis in Preterm Neonates Born at < 32 Weeks' Gestation." Am J Perinatol **32**(7): 675-682.

Smit, A. L., J. V. Been, L. J. Zimmermann, R. F. Kornelisse, P. Andriessen, S. F. Vanterpool, M. P. Bischoff, R. J. Stokroos, R. R. de Krijger, B. Kremer and B. W. Kramer (2015). "Automated auditory brainstem response in preterm newborns with histological chorioamnionitis." J Matern Fetal Neonatal Med **28**(15): 1864-1869.
Smulian, J. C., S. Shen-Schwarz, A. M. Vintzileos, M. F. Lake and C. V. Ananth (1999). "Clinical chorioamnionitis and histologic placental inflammation." Obstet Gynecol **94**(6): 1000-1005.
Soraisham, A. S., N. Singhal, D. D. McMillan, R. S. Sauve, S. K. Lee and N. Canadian Neonatal (2009). "A multicenter study on the clinical outcome of chorioamnionitis in preterm infants." Am J Obstet Gynecol **200**(4): 372 e371-376.
Soraisham, A. S., C. Trevenen, S. Wood, N. Singhal and R. Sauve (2013). "Histological chorioamnionitis and neurodevelopmental outcome in preterm infants." J Perinatol **33**(1): 70-75.
Stepan, M., T. Cobo, J. Maly, M. Navratilova, I. Musilova, H. Hornychova, B. Jacobsson and M. Kacerovsky (2016). "Neonatal outcomes in subgroups of women with preterm prelabor rupture of membranes before 34 weeks." J Matern Fetal Neonatal Med **29**(14): 2373-2377.
Stimac, M., E. Juretic, V. Vukelic, N. P. Matasic, M. Kos and D. Babic (2014). "Effect of chorioamnionitis on mortality, early onset neonatal sepsis and bronchopulmonary dysplasia in preterm neonates with birth weight of < 1,500 grams." Coll Antropol **38**(1): 167-171.
Stranak, Z., J. Feyereisl, P. Korcek, S. Feyereislova and L. Krofta (2016). "Procalcitonin is more likely to be released by the fetus rather than placental tissue during chorioamnionitis." Biomed Pap Med Fac Univ Palacky Olomouc Czech Repub **160**(4): 499-502.
Strunk, T., D. Doherty, A. Jacques, K. Simmer, P. Richmond, R. Kohan, A. Charles and D. Burgner (2012). "Histologic chorioamnionitis is associated with reduced risk of late-onset sepsis in preterm infants." Pediatrics **129**(1): e134-141.
Sung, J. H., S. J. Choi, S. Y. Oh, C. R. Roh and J. H. Kim (2017). "Revisiting the diagnostic criteria of clinical chorioamnionitis in preterm birth." BJOG **124**(5): 775-783.
Trevisanuto, D., C. Peruzzetto, F. Cavallin, S. Vedovato, E. Cosmi, S. Visentin, S. Chiarelli and V. Zanardo (2013). "Fetal placental inflammation is associated with poor neonatal growth of preterm infants: a case-control study." J Matern Fetal Neonatal Med **26**(15): 1484-1490.
Tsai, C. H., Y. Y. Chen, K. G. Wang, C. Y. Chen and C. P. Chen (2012). "Characteristics of early-onset neonatal sepsis caused by Escherichia coli." Taiwan J Obstet Gynecol **51**(1): 26-30.
Tsiartas, P., M. Kacerovsky, I. Musilova, H. Hornychova, T. Cobo, K. Savman and B. Jacobsson (2013). "The association between histological chorioamnionitis, funisitis and neonatal outcome in women with preterm prelabor rupture of membranes." J Matern Fetal Neonatal Med **26**(13): 1332-1336.
Tudela, C. M., R. D. Stewart, S. W. Roberts, G. D. Wendel, Jr., I. A. Stafford, D. D. McIntire and J. S. Sheffield (2012). "Intrapartum evidence of early-onset group B streptococcus." Obstet Gynecol **119**(3): 626-629.
Van Marter, L. J., O. Dammann, E. N. Allred, A. Leviton, M. Pagano, M. Moore, C. Martin and I. Developmental Epidemiology Network (2002). "Chorioamnionitis, mechanical ventilation, and postnatal sepsis as modulators of chronic lung disease in preterm infants." J Pediatr **140**(2): 171-176.
van Vliet, E. O., J. F. de Kieviet, J. P. van der Voorn, J. V. Been, J. Oosterlaan and R. M. van Elburg (2012). "Placental pathology and long-term neurodevelopment of very preterm infants." Am J Obstet Gynecol **206**(6): 489 e481-487.
Vander Haar, E. and C. Gyamfi-Bannerman (2016). "Chorioamnionitis and Neurocognitive Development at Age 2 Years." Obstet Gynecol **127**(3): 437-441.
Vinnars, M. T., N. Papadogiannakis, J. Nasiell, G. Holmstrom and M. Westgren (2015). "Placental pathology in relation to stillbirth and neonatal outcome in an extremely preterm population: a prospective cohort study." Acta Obstet Gynecol Scand **94**(6): 584-590.
Watterberg, K. L., J. S. Gerdes, K. L. Gifford and H. M. Lin (1999). "Prophylaxis against early adrenal insufficiency to prevent chronic lung disease in premature infants." Pediatrics **104**(6): 1258-1263.
Wynn, J. L., N. I. Hansen, A. Das, C. M. Cotten, R. N. Goldberg, P. J. Sanchez, E. F. Bell, K. P. Van Meurs, W. A. Carlo, A. R. Laptook, R. D. Higgins, D. K. Benjamin, Jr., B. J. Stoll, H. Eunice Kennedy Shriver National Institute of Child and N. Human Development Neonatal Research (2013). "Early sepsis does not increase the risk of late sepsis in very low birth weight neonates." J Pediatr **162**(5): 942-948 e941-943.
Xie, A., W. Zhang, M. Chen, Y. Wang, Y. Wang, Q. Zhou and X. Zhu (2015). "Related factors and adverse neonatal outcomes in women with preterm premature rupture of membranes complicated by histologic chorioamnionitis." Med Sci Monit **21**: 390-395.
Yancey, M. K., P. Duff, P. Kubilis, P. Clark and B. H. Frentzen (1996). "Risk factors for neonatal sepsis." Obstet Gynecol **87**(2): 188-194.

Yoon, B. H., R. Romero, C. J. Kim, J. K. Jun, R. Gomez, J. H. Choi and H. C. Syn (1995). "Amniotic fluid interleukin-6: a sensitive test for antenatal diagnosis of acute inflammatory lesions of preterm placenta and prediction of perinatal morbidity." Am J Obstet Gynecol **172**(3): 960-970.

Supplementary Table 2. Meta-regressions

Meta-regression		k	CC	95% CI	Z	P	R^2
Early onset sepsis	Gestational age (MD, weeks)	40	-0.07	-0.28 to 0.13	-0.69	0.490	0.00
	Birth weight (MD, per 100 g)	40	-0.04	-0.15 to 0.07	-0.74	0.457	0.00
	CA type (histological vs. clinical)	65	0.32	-0.02 to 0.66	1.83	0.068	0.08
	ACS (log OR)	20	0.17	-0.18 to 0.51	0.96	0.338	0.00
	Cesarean section (log OR)	24	0.09	-0.11 to 0.28	0.86	0.390	0.00
	SGA (log OR)	12	0.53	-0.08 to 1.14	1.70	0.088	0.32
	PROM (log OR)	18	0.17	-0.31 to 0.66	0.70	0.483	0.13
Late onset sepsis	Gestational age (MD, weeks)	22	-0.33	-0.50 to -0.17	-3.89	< 0.001	0.54
	Birth weight (MD, per 100 g)	23	-0.09	-0.21 to 0.03	-1.48	0.138	0.00
	CA type (histological vs. clinical)	27	-0.13	-0.45 to 0.18	-0.81	0.415	0.00
	ACS (log OR)	18	-0.18	-0.39 to 0.02	-1.79	0.073	0.36
	Cesarean section (log OR)	17	-0.11	-0.33 to 0.10	-1.04	0.300	0.23
	SGA (log OR)	11	0.20	-0.03 to 0.42	1.73	0.083	0.10
	PROM (log OR)	14	-0.20	-0.54 to 0.14	-1.15	0.251	0.26

MD: mean difference; CA: chorioamnionitis; ACS: antenatal corticosteroids; SGA: small for gestational age; PROM: preterm rupture of membranes.

1.2 Supplementary Figures

Study name	Odds ratio	Lower limit	Upper limit	p-Value
	Statistics for each study			
Kim 2015	0.78	0.07	8.78	0.844
Kacerovsky 2014	1.02	0.06	16.79	0.988
Dulay 2008	1.04	0.18	5.86	0.969
Averbuch 1995	1.19	0.28	4.97	0.813
Been 2010	1.26	0.53	3.02	0.602
Cuna 2014	1.35	0.12	15.06	0.807
Lee S.Y. 2012	1.96	0.91	4.19	0.084
Arayici 2014	2.02	1.21	3.38	0.007
Buhimschi 2007	2.20	0.60	8.03	0.235
Strunk 2012	2.31	1.03	5.15	0.041
Stimac 2014	2.91	1.81	4.68	0.000
Buhimschi 2009	3.43	0.19	62.81	0.406
Lau 2005	3.98	1.66	9.57	0.002
Jeon 2014	4.49	2.06	9.80	0.000
Ahn 2012	4.94	0.94	26.00	0.059
Ogunyemi 2003	5.12	2.31	11.34	0.000
Park 2017	5.37	0.98	29.46	0.053
Wynn 2013	5.76	3.71	8.92	0.000
Popowski 2011	7.47	2.56	21.81	0.000
Dempsey 2005	7.93	2.23	28.16	0.001
Hendson 2011	8.98	2.05	39.40	0.004
Stepan 2016	9.19	0.49	172.95	0.138
Liu 2014	10.07	0.54	189.45	0.123
Arora 2015	10.39	3.23	33.43	0.000
Lee S.Y.R. 2012	11.76	0.60	230.23	0.104
Park 2004	14.00	1.66	118.31	0.015
Ohyama 2002	25.48	1.50	433.05	0.025
**Histological CA**	**3.52**	**2.68**	**4.63**	**0.000**

Heterogeneity: I^2 = 34%; p = 0.041

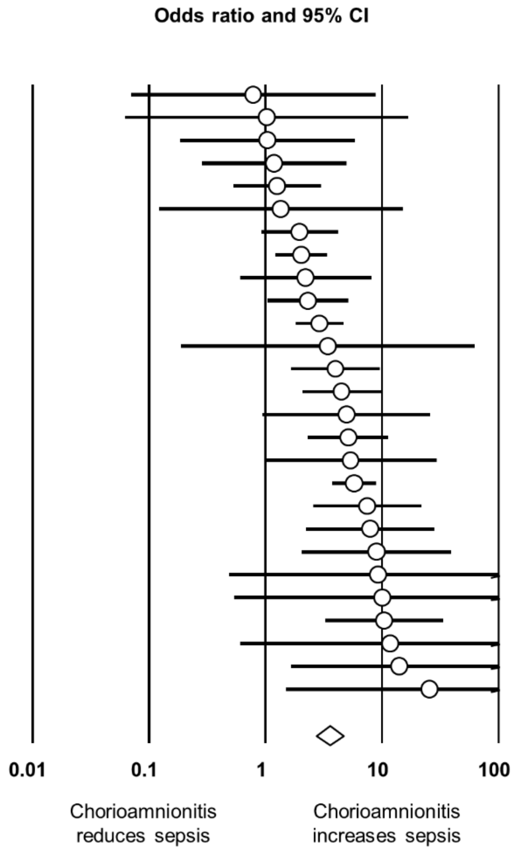

Supplementary Figure 1. Random effects meta-analysis of histological chorioamnionitis (CA) and culture proven early onset sepsis.

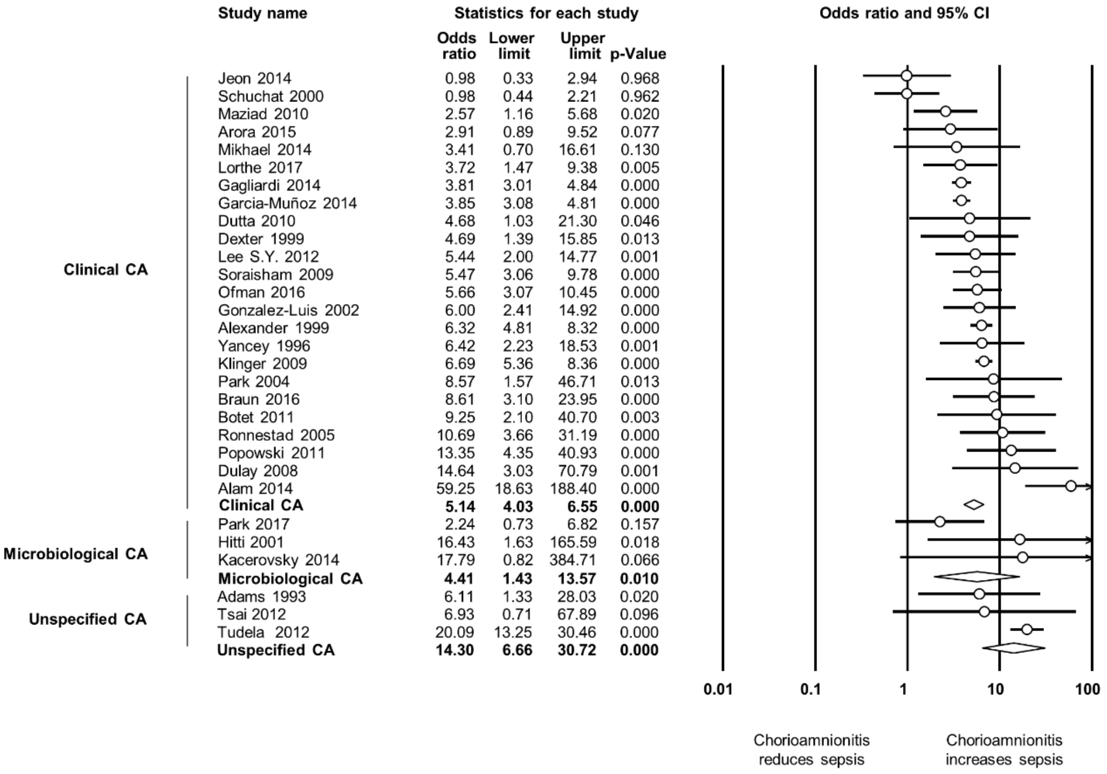

Supplementary Figure 2. Random effects meta-analyses of clinical, microbiological, and unspecified chorioamnionitis (CA) and culture proven early onset sepsis. Microbiological CA: microbial invasion of the amniotic cavity.

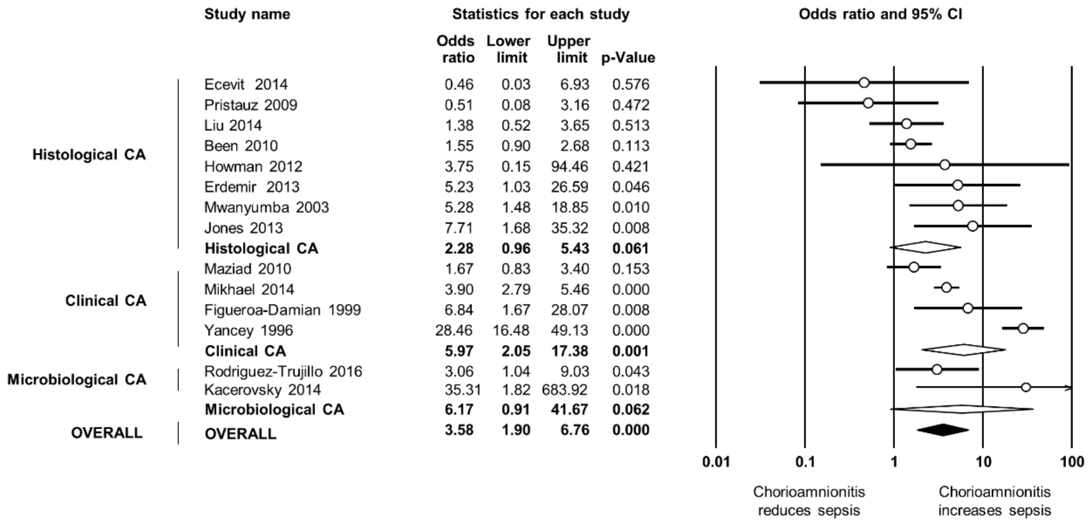

Supplementary Figure 3. Random effects meta-analysis of histological, clinical and microbiological chorioamnionitis (CA) and clinical early onset sepsis. Microbiological CA: microbial invasion of the amniotic cavity.

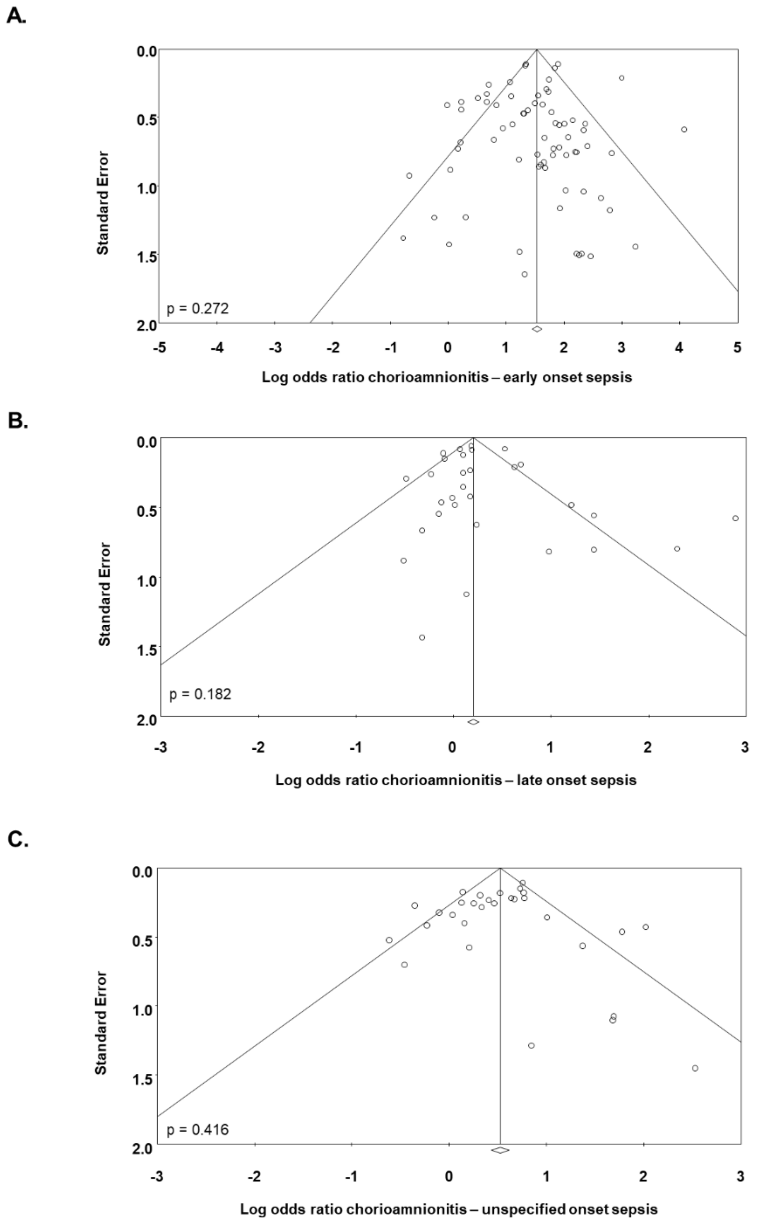

Supplementary Figure 4. Funnel plots for publication bias and results of Egger's regression test. Publication bias plot for (A) chorioamnionitis and early onset sepsis, (B) chorioamnionitis and late onset sepsis, and (C) chorioamnionitis and unspecified onset sepsis.

Group	Study name	Odds ratio	Lower limit	Upper limit	p-Value
Histological CA	Stepan 2016	0.73	0.04	12.08	0.823
	Choi 2008	0.73	0.20	2.68	0.630
	Kim 2015	0.88	0.36	2.20	0.791
	Strunk 2012	0.91	0.68	1.24	0.562
	Chau 2012	0.99	0.42	2.33	0.985
	Ahn 2012	1.11	0.55	2.22	0.774
	Been 2010	1.11	0.68	1.82	0.681
	Liu 2014	1.19	0.52	2.74	0.682
	Ogunyemi 2003	1.87	1.24	2.82	0.003
	Lau 2005	1.99	1.36	2.92	0.000
	Mu 2008	3.35	1.30	8.65	0.013
	Puri 2016	4.22	0.87	20.59	0.075
	**Histological CA**	1.34	1.07	1.68	0.011
Clinical CA	Botet 2011	1.19	0.75	1.88	0.461
	Garcia-Muñoz 2014	1.20	1.07	1.35	0.002
	Shah 2015	1.21	1.01	1.44	0.033
	Gagliardi 2014	1.69	1.44	1.99	0.000
	**Clinical CA**	1.32	1.05	1.67	0.017
Microbiological CA	Kirchner 2007	9.90	2.07	47.49	0.004
	**Microbiological CA**	**9.90**	**1.96**	**49.98**	**0.005**
Unspecified CA	Alshaikh 2014	0.61	0.35	1.10	0.099
	**Unspecified CA**	**0.61**	**0.30**	**1.24**	**0.176**
**OVERALL**	**OVERALL**	**1.31**	**1.12**	**1.53**	**0.001**

Heterogeneity: I^2 = 64%; p < 0.001

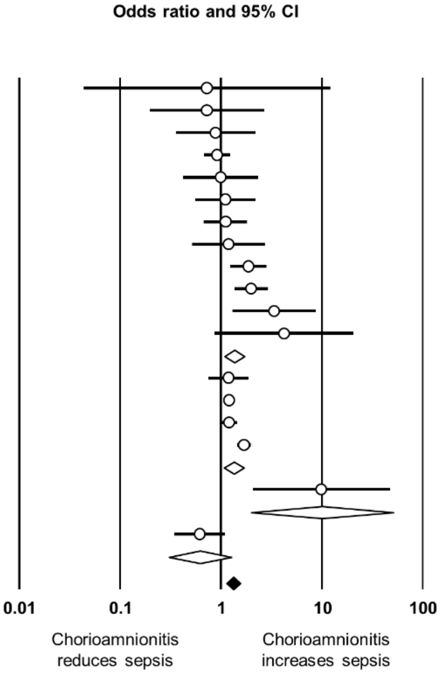

Supplementary Figure 5. Random effects meta-analyses of histological, clinical, microbiological, and unspecified chorioamnionitis (CA) and culture proven late onset sepsis. Microbiological CA: microbial invasion of the amniotic cavity.

Study name	Statistics for each study			
	Odds ratio	Lower limit	Upper limit	p-Value
Been 2010	0.80	0.48	1.33	0.383
Ecevit 2014	1.14	0.13	10.39	0.906
Liu 2014	1.37	0.64	2.94	0.422
Erdemir 2013	2.67	0.54	13.26	0.231
Howman 2012	4.21	1.41	12.62	0.010
**OVERALL**	1.52	0.78	2.96	0.216

Heterogeneity: I^2 = 53%; p = 0.073

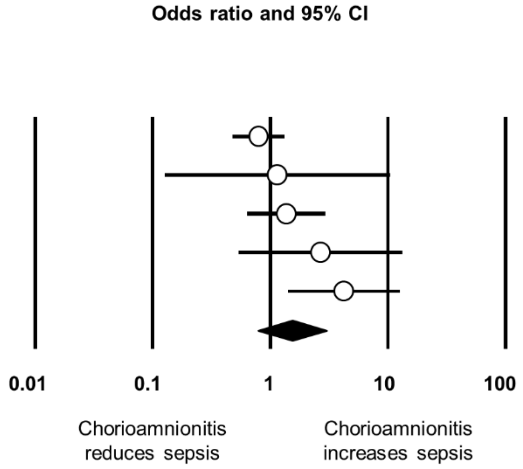

Supplementary Figure 6. Random effects meta-analyses of histological chorioamnionitis (CA) and clinical late onset sepsis.

Group	Study name	Odds ratio	Lower limit	Upper limit	p-Value
Histological CA	Van Vliet 2012	0.54	0.19	1.51	0.239
	Van Marter 2002	0.70	0.41	1.21	0.203
	Prendergast 2011	0.80	0.35	1.81	0.586
	Sato 2011	0.90	0.48	1.71	0.756
	Vinnars 2015	1.04	0.53	2.03	0.915
	Plakkal 2013	1.04	0.68	1.60	0.845
	Soraisham 2013	1.14	0.69	1.87	0.615
	Smit 2015	1.15	0.82	1.63	0.420
	Jessop 2016	1.17	0.53	2.57	0.692
	Watterberg 1999	1.23	0.40	3.82	0.720
	Van Marter 2002	1.39	0.59	3.29	0.446
	Dessardo 2014	1.59	0.96	2.64	0.073
	Rocha 2006	1.96	1.25	3.06	0.003
	Lahra 2009	2.08	1.55	2.79	0.000
	Miyazaki 2016	2.13	1.72	2.64	0.000
	Elimian 2000	2.15	1.50	3.09	0.000
	Bordigato 2011	2.33	0.19	29.04	0.510
	Smulian 1999	2.74	1.35	5.56	0.005
	Mehta 2006	3.94	1.30	11.96	0.015
	Dexter 2000	5.44	0.66	44.80	0.115
	Rao 2001	5.93	2.38	14.74	0.000
	Guzick 1985	7.55	3.25	17.55	0.000
	Dollner 2002	12.55	0.73	216.03	0.081
	**Histological**	1.60	1.30	1.97	0.000
Clinical CA	Aziz 2009	1.29	0.77	2.15	0.330
	Vander Haar 2016	1.38	0.93	2.04	0.110
	Austeng 2010	1.40	0.80	2.45	0.239
	Ballard 2016	1.50	0.94	2.39	0.085
	Rouse 2004	1.90	1.23	2.93	0.004
	Sung 2017	2.17	1.40	3.35	0.000
	Churgay 1994	5.38	0.62	46.95	0.128
	**Clinical**	1.63	1.16	2.28	0.005
Both CA	Schlapbach 2010	0.63	0.16	2.52	0.516
	**Both**	0.63	0.14	2.97	0.562
Unspecified CA	Gyamfi-Bannerman 2014	1.69	1.18	2.42	0.004
	**Unspecified**	1.69	0.77	3.70	0.190
OVERALL	**OVERALL**	1.59	1.34	1.89	0.000

Heterogeneity: I^2 = 61%; $p < 0.001$

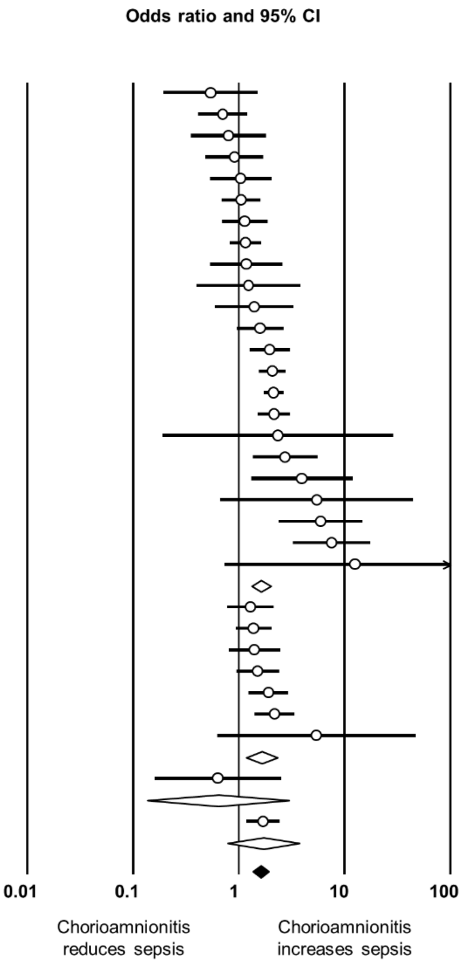

Supplementary Figure 7. Random effects meta-analyses of histological, clinical, both (histological and clinical CA), and unspecified chorioamnionitis (CA) and all (culture proven or clinical) unspecified onset sepsis.

Group	Study name	Odds ratio	Lower limit	Upper limit	p-Value
Histological CA	Van Vliet 2012	0.54	0.19	1.51	0.239
	Van Marter 2002	0.70	0.41	1.21	0.203
	Sato 2011	0.90	0.48	1.71	0.756
	Vinnars 2015	1.04	0.53	2.03	0.915
	Plakkal 2013	1.04	0.68	1.60	0.845
	Soraisham 2013	1.14	0.69	1.87	0.615
	Smit 2015	1.15	0.82	1.63	0.420
	Watterberg 1999	1.23	0.40	3.82	0.720
	Rocha 2006	1.96	1.25	3.06	0.003
	Lahra 2009	2.08	1.55	2.79	0.000
	Miyazaki 2016	2.13	1.72	2.64	0.000
	Elimian 2000	2.15	1.50	3.09	0.000
	Bordigato 2011	2.33	0.19	29.04	0.510
	Mehta 2006	3.94	1.30	11.96	0.015
	Dollner 2002	12.55	0.73	216.03	0.081
	**Histological**	**1.42**	**1.14**	**1.78**	**0.002**
Clinical CA	Aziz 2009	1.29	0.77	2.15	0.330
	Vander Haar 2016	1.38	0.93	2.04	0.110
	Ballard 2016	1.50	0.94	2.39	0.085
	Rouse 2004	1.90	1.23	2.93	0.004
	Sung 2017	2.17	1.40	3.35	0.000
	**Clinical**	**1.62**	**1.16**	**2.27**	**0.005**
Both CA	Schlapbach 2010	0.63	0.16	2.52	0.516
	**Both**	**0.63**	**0.14**	**2.86**	**0.552**
Unspecified CA	Gyamfi-Bannerman 2014	1.69	1.18	2.42	0.004
	**Unspecified**	**1.69**	**0.83**	**3.42**	**0.145**
OVERALL	**OVERALL**	**1.48**	**1.23**	**1.76**	**0.000**

Heterogeneity: I^2 = 59%; p < 0.001

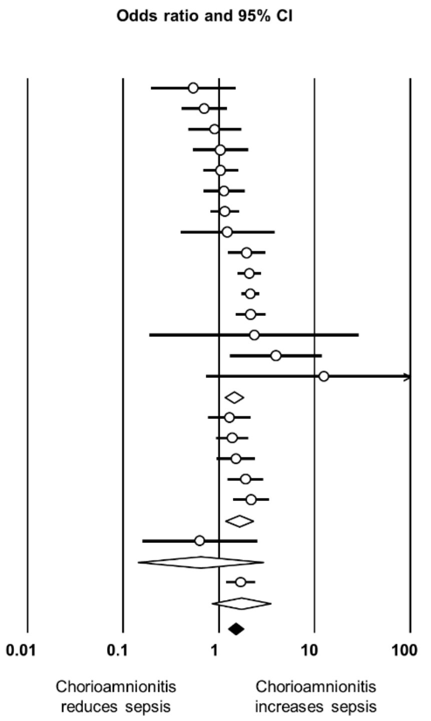

Supplementary Figure 8. Random effects meta-analysis of histological, clinical, both (histological and clinical CA), and unspecified chorioamnionitis (CA) and culture proven unspecified onset sepsis.

Group	Study name	Odds ratio	Lower limit	Upper limit	p-Value
Histological	Jessop 2016	1.17	0.53	2.57	0.692
	Van Marter 2002	1.39	0.59	3.29	0.446
	Dessardo 2014	1.59	0.96	2.64	0.073
	Smulian 1999	2.74	1.35	5.56	0.005
	Rao 2001	5.93	2.38	14.74	0.000
	**Histological**	**2.04**	**1.23**	**3.37**	**0.006**
Clinical	Churgay 1994	5.38	0.62	46.95	0.128
	**Clinical**	**5.38**	**0.53**	**55.04**	**0.156**
OVERALL	**OVERALL**	**2.13**	**1.30**	**3.48**	**0.003**

Heterogeneity: I^2 = 51%; p = 0.069

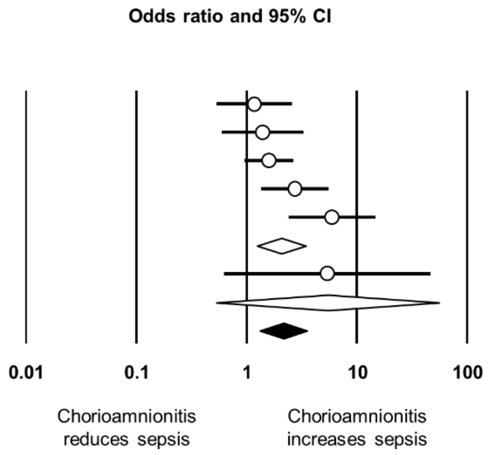

Supplementary Figure 9. Random effects meta-analysis of histological and clinical chorioamnionitis and clinical unspecified onset sepsis.

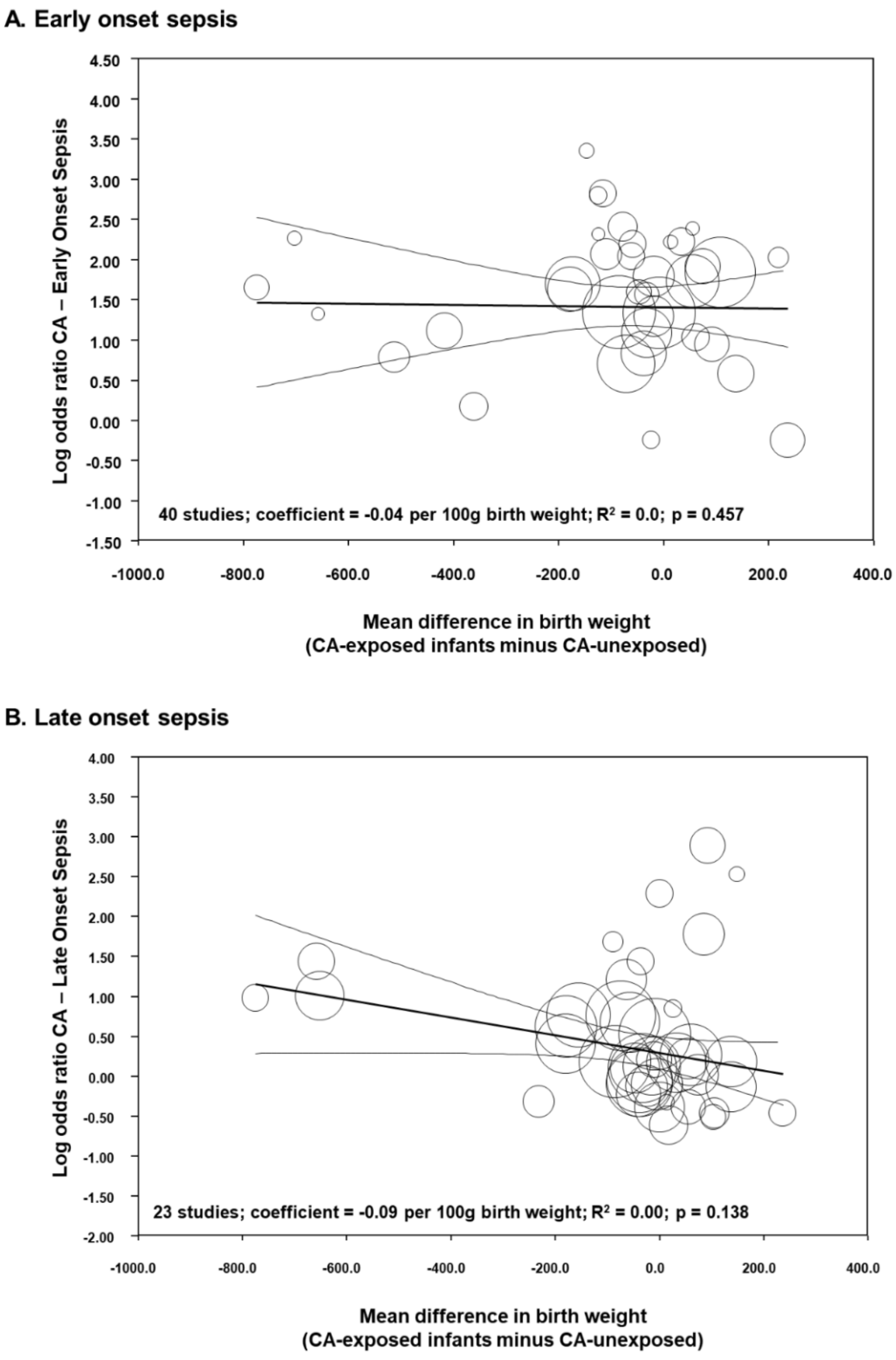

Supplementary Figure 10. Meta-regression plot of association between chorioamnionitis and (A) early onset sepsis, and (B) late onset sepsis controlling for difference in birth weight between exposed and non-exposed groups.

Study name	Odds ratio	Lower limit	Upper limit	p-Value
**Non-significant difference in GA (p ≥ 0.05)**				
Kacerovsky 2014	1.02	0.06	16.79	0.988
Nasef 2013	1.24	0.32	4.74	0.754
Trevisanuto 2013	1.26	0.58	2.70	0.559
Arayici 2014	2.02	1.21	3.38	0.007
Janota 2001	4.80	0.89	25.96	0.069
Ahn 2012	4.94	0.94	26.00	0.059
Ofman 2016	5.66	3.07	10.45	0.000
Gonzalez-Luis 2002	6.00	2.41	14.92	0.000
Barrera-Reyes 2011	6.81	2.29	20.30	0.001
Stepan 2016	9.19	0.49	172.95	0.138
Botet 2011	9.25	2.10	40.70	0.003
Liu 2014	10.07	0.54	189.45	0.123
Stranak 2016	11.11	2.76	44.77	0.001
Lee S.Y. 2012	11.76	0.60	230.23	0.104
Ohyama 2002	25.48	1.50	433.05	0.025
**Non-sign. difference in GA**	**3.76**	**2.66**	**5.32**	**0.000**
**Significant difference in GA (p < 0.05)**				
Kim 2015	0.78	0.07	8.78	0.844
Averbuch 1995	1.19	0.28	4.97	0.813
Been 2010	1.26	0.53	3.02	0.602
Buhimschi 2007	2.20	0.60	8.03	0.235
Strunk 2012	2.31	1.03	5.15	0.041
Rodriguez-Trujillo 2016	3.06	1.04	9.03	0.043
Alexander 1998	3.66	1.45	9.22	0.006
Howman 2012	3.75	0.15	94.46	0.421
Gagliardi 2014	3.81	3.01	4.84	0.000
Garcia-Muñoz 2014	3.85	3.08	4.81	0.000
Lau 2005	3.98	1.66	9.57	0.002
Pappas 2013	4.74	2.42	9.26	0.000
Ogunyemi 2003	5.12	2.31	11.34	0.000
Erdemir 2013	5.23	1.03	26.59	0.046
Park 2017	5.37	0.98	29.46	0.053
Soraisham 2009	5.47	3.06	9.78	0.000
Jones 2013	7.71	1.68	35.32	0.008
Dempsey 2005	7.93	2.23	28.16	0.001
Hendson 2011	8.98	2.05	39.40	0.004
Yoon 1995	9.65	0.50	184.88	0.132
Hitti 2001	16.43	1.63	165.59	0.018
De Felice 2005	16.87	3.78	75.32	0.000
**Significant difference in GA**	**3.95**	**3.11**	**5.02**	**0.000**
**OVERALL**	**3.89**	**3.19**	**4.73**	**0.000**

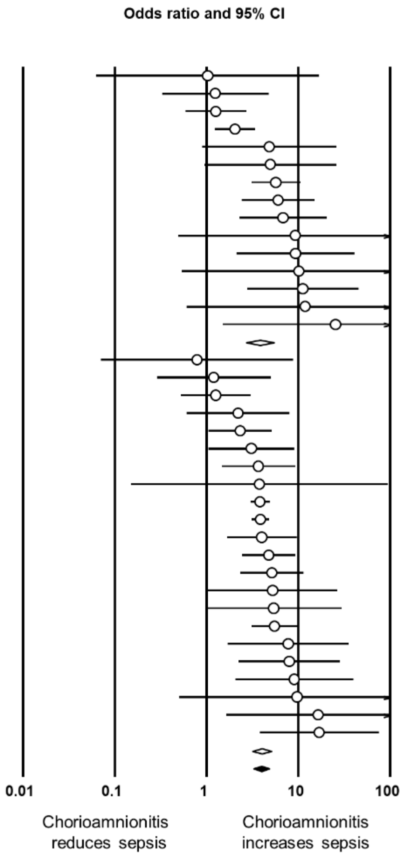

Supplementary Figure 11. Random effects meta-analysis of chorioamnionitis and early onset sepsis, comparing studies where the mean difference in GA in the chorioamnionitis group was significantly different ($p < 0.05$), and studies where it was non-significant ($p \geq 0.05$)

Group	Study name	Odds ratio	Lower limit	Upper limit	p-Value
**Non-significant difference in GA (p ≥ 0.05)**	Bry 2015	0.60	0.11	3.40	0.564
	Stepan 2016	0.73	0.04	12.08	0.823
	Trevisanuto 2013	0.86	0.29	2.52	0.785
	Barrera-Reyes 2011	1.02	0.39	2.62	0.974
	Ahn 2012	1.11	0.55	2.22	0.774
	Botet 2011	1.19	0.75	1.88	0.461
	Liu 2014	1.19	0.52	2.74	0.682
	**Non-sign. difference in GA**	**1.06**	**0.72**	**1.57**	**0.755**
**Significant difference in GA (p < 0.05)**	Choi 2008	0.73	0.20	2.68	0.630
	Been 2010	0.80	0.48	1.33	0.383
	Kim 2015	0.88	0.36	2.20	0.791
	Pappas 2013	0.90	0.72	1.13	0.369
	Strunk 2012	0.91	0.68	1.24	0.562
	Pappas 2013	1.07	0.91	1.26	0.420
	Been 2010	1.11	0.68	1.82	0.681
	Garcia-Muñoz 2014	1.20	1.07	1.35	0.002
	Gagliardi 2014	1.69	1.44	1.99	0.000
	Ogunyemi 2003	1.87	1.24	2.82	0.003
	Lau 2005	1.99	1.36	2.92	0.000
	Erdemir 2013	2.67	0.54	13.26	0.231
	Mu 2008	3.35	1.30	8.65	0.013
	Howman 2012	4.21	1.41	12.62	0.010
	Puri 2016	4.22	0.87	20.59	0.075
	Kirchner 2007	9.90	2.07	47.49	0.004
	**Significant difference in GA**	**1.33**	**1.11**	**1.60**	**0.002**
	**OVERALL**	**1.28**	**1.09**	**1.51**	**0.003**

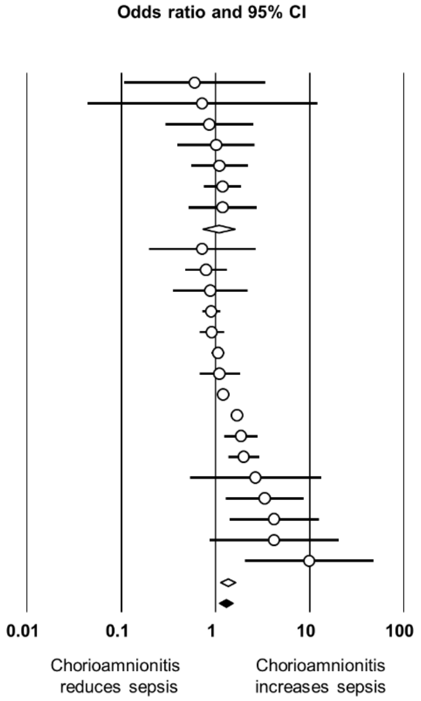

Supplementary Figure 12. Random effects meta-analysis of chorioamnionitis and late onset sepsis, comparing studies where the mean difference in GA in the chorioamnionitis group was significantly different ($p < 0.05$), and studies where it was non-significant ($p \geq 0.05$).

	Study name	Odds ratio	Lower limit	Upper limit	p-Value
Adjusted data	Ahn 2012	2.50	0.26	24.01	0.427
	Alam 2014	4.10	0.61	27.40	0.145
	Alexander 1998	2.70	1.03	7.09	0.044
	Been 2010	2.20	1.01	4.79	0.047
	Braun 2016	8.70	3.09	24.51	0.000
	Dutta 2010	8.84	1.81	43.17	0.007
	Gagliardi 2014	0.25	0.19	0.33	0.000
	Garcia-Muñoz 2014	3.10	2.31	4.17	0.000
	Lorthe 2017	1.00	0.98	1.02	1.000
	Nasef 2013	0.70	0.16	3.06	0.636
	Ofman 2016	4.10	2.83	5.94	0.000
	Oh 2015	1.42	0.22	9.27	0.714
	Park 2004	3.90	0.29	52.37	0.304
	Ronnestad 2005	10.50	3.30	33.40	0.000
	Soraisham 2009	5.54	2.87	10.69	0.000
	**All adjusted data**	**2.51**	**1.51**	**4.14**	**0.000**
Unadjusted data	Ahn 2012	4.94	0.94	26.00	0.059
	Alam 2014	59.25	18.63	188.40	0.000
	Alexander 1998	3.66	1.45	9.22	0.006
	Been 2010	1.26	0.53	3.02	0.602
	Braun 2016	8.61	3.10	23.95	0.000
	Dutta 2010	4.68	1.03	21.30	0.046
	Gagliardi 2014	3.81	3.01	4.84	0.000
	Garcia-Muñoz 2014	3.85	3.08	4.81	0.000
	Lorthe 2017	3.72	1.47	9.38	0.005
	Nasef 2013	1.24	0.32	4.74	0.754
	Ofman 2016	5.66	3.07	10.45	0.000
	Park 2004	14.00	1.66	118.31	0.015
	Ronnestad 2005	10.69	3.66	31.19	0.000
	Soraisham 2009	5.47	3.06	9.78	0.000
	**All unadjusted data**	**5.10**	**3.07**	**8.46**	**0.000**

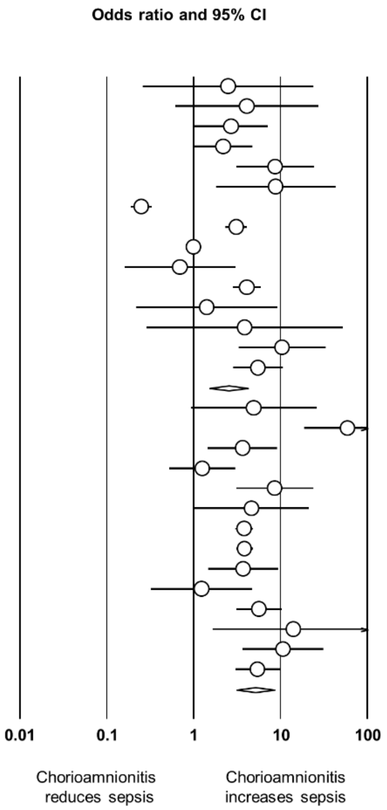

Supplementary Figure 13. Random effects meta-analysis of studies with adjusted results for chorioamnionitis and early onset sepsis, comparing adjusted and unadjusted results.

Group	Study name	Odds ratio	Lower limit	Upper limit	p-Value
Adjusted data	Ahn 2012	0.32	0.10	1.04	0.057
	Gagliardi 2014	1.19	0.94	1.50	0.145
	Garcia-Muñoz 2014	0.85	0.73	0.99	0.035
	Lau 2005	1.90	1.10	3.29	0.022
	Mu 2008	3.36	1.28	8.83	0.014
	Oh 2015	0.96	0.39	2.40	0.934
	Strunk 2012	0.75	0.58	0.97	0.028
	**All adjusted data**	**1.04**	**0.79**	**1.38**	**0.770**
Unadjusted data	Ahn 2012	1.11	0.55	2.22	0.774
	Gagliardi 2014	1.69	1.44	1.99	0.000
	Garcia-Muñoz 2014	1.20	1.07	1.35	0.002
	Lau 2005	1.99	1.36	2.92	0.000
	Mu 2008	3.35	1.30	8.65	0.013
	Strunk 2012	0.91	0.68	1.24	0.562
	**All unadjusted data**	**1.42**	**1.08**	**1.87**	**0.012**

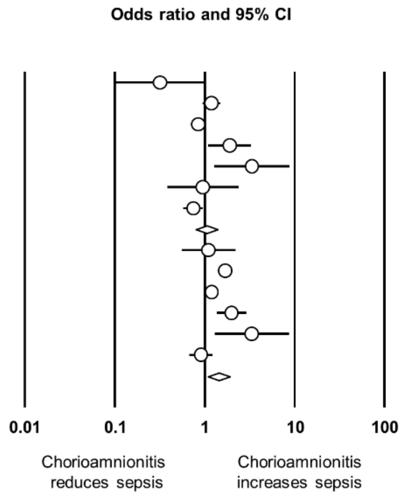

Supplementary Figure 14. Random effects meta-analysis of studies with adjusted results for chorioamnionitis and late onset sepsis, comparing adjusted and unadjusted results.

1.3 Data File 1. Details of systematic search.

**Search Criteria #1: Intervention/Exposure**		
**Mesh-terms**	**Free-Text**	
"Chorioamnionitis"[Mesh]	Chrorioamnionitis<br>Chorioamnionitis, acute<br>Acute Chorioamnionitis<br>Chorioamnionitides<br>Amnionitis	Amnionitides<br>Funisitis<br>Funisitides<br>Intrauterine infection<br>Intra-uterine infection<br>Placental inflammation
**Search String**		
((((((((((("Chorioamnionitis"[Mesh]) OR Chrorioamnionitis) OR Chorioamnionitis, acute) OR Acute Chorioamnionitis) OR Chorioamnionitides) OR Amnionitis) OR Amnionitides) OR Funisitis) OR Funisitides) OR Intrauterine infection) OR Intra-uterine infection) OR Placental inflammation		

**Search Criteria #2: Disease of Interest/Health problem**			
**Mesh-terms**	**Free-Text**		
"Neonatal Sepsis"[Mesh]<br><br>"Sepsis"[Mesh]	Neonatal Sepsis<br><br>Neonatal Sepses<br><br>Sepses, Neonatal<br>Sepsis, Neonatal<br><br>Neonatal Late-Onset Sepsis<br><br>Late-Onset Sepses, Neonatal<br><br>Late-Onset Sepsis, Neonatal<br>Neonatal Late Onset Sepsis<br><br>Neonatal Late-Onset Sepses<br>Sepses, Neonatal Late-Onset<br><br>Sepsis, Neonatal Late-Onset	Neonatal Early Onset Sepsis<br><br>Neonatal Early-Onset Sepses<br><br>Neonatal Early-Onset Sepsis<br><br>Early-Onset Sepses, Neonatal<br><br>Early-Onset Sepsis, Neonatal<br>Septicemia<br>Septicemias<br>Poisoning, Blood<br>Blood Poisoning<br>Blood Poisonings<br>Poisonings, Blood	Sepses, Neonatal Early-Onset<br><br>Sepsis, Neonatal Early-Onset<br>Sepsis<br>Severe Sepsis<br>Sepsis, Severe<br>Pyemia<br>Pyemias<br>Pyohemia<br>Pyohemias<br>Pyaemia<br>Pyaemias
**Search String**			
((((((((((((((((((((((((((((((((((("Neonatal Sepsis"[Mesh]) OR "Sepsis"[Mesh]) OR Neonatal Sepsis) OR Neonatal Sepses) OR Sepses, Neonatal) OR Sepsis, Neonatal) OR Neonatal Late-Onset Sepsis) OR Late-Onset Sepses, Neonatal) OR Late-			

Onset Sepsis, Neonatal) OR Neonatal Late Onset Sepsis) OR Neonatal Late-Onset Sepses) OR Sepses, Neonatal Late-Onset) OR Sepsis, Neonatal Late-Onset) OR Neonatal Early-Onset Sepsis) OR Early-Onset Sepses, Neonatal) OR Early-Onset Sepsis, Neonatal) OR Neonatal Early Onset Sepsis) OR Neonatal Early-Onset Sepses) OR Sepses, Neonatal Early-Onset) OR Sepsis, Neonatal Early-Onset) OR Sepsis) OR Severe Sepsis) OR Sepsis, Severe) OR Pyemia) OR Pyemias) OR Pyohemia) OR Pyohemias) OR Pyaemia) OR Pyaemias) OR Septicemia) OR Septicemias) OR Poisoning, Blood) OR Blood Poisoning) OR Blood Poisonings)

**Search Criteria #3: Study Population**			
**Mesh-terms**	**Free-Text**		
"Infant, Premature"[Mesh]<br>"Premature Birth"[Mesh]<br>"Infant, Extremely Premature"[Mesh]	Infants, Premature<br>Premature Infant<br><br>Preterm Infants<br><br>Infant, Preterm<br><br>Infants, Preterm<br><br>Preterm Infant<br><br>Premature Infants<br><br>Neonatal Prematurity<br><br>Prematurity, Neonatal<br><br>Neonate, preterm<br><br>Neonates, preterm	Preterm neonates<br><br>Preterm neonate<br>Birth, Premature<br><br>Births, Premature<br><br>Premature Births<br>Preterm Birth<br><br>Birth, Preterm<br><br>Births, Preterm<br><br>Preterm Births<br><br>Extremely Premature Infant	Infants, Extremely Premature<br><br>Premature Infant, Extremely<br><br>Premature Infants, Extremely<br>Extremely Preterm Infants<br><br>Extremely Preterm Infant<br><br>Infant, Extremely Preterm<br><br>Infants, Extremely Preterm<br><br>Preterm Infant, Extremely<br><br>Preterm Infants, Extremely<br><br>Extremely Premature Infants
**Search String**	**Hits: 117990**		
**((((((((((((((((((((((((((((((((("Infant, Premature"[Mesh]) OR "Premature Birth"[Mesh]) OR "Infant, Extremely Premature"[Mesh]) OR Infants, Premature) OR Premature Infant) OR Preterm Infants) OR Infant, Preterm) OR Infants, Preterm) OR Preterm Infant) OR Premature Infants) OR Neonatal Prematurity) OR Prematurity, Neonatal) OR Neonate, preterm) OR Neonates, preterm) OR Preterm neonates) OR Preterm neonate) OR Birth, Premature) OR Births, Premature) OR Premature Births) OR Preterm Birth) OR Birth, Preterm) OR Births, Preterm) OR Preterm Births) OR Extremely Premature Infant) OR Infants, Extremely Premature) OR Premature Infant, Extremely) OR Premature Infants, Extremely) OR Extremely Preterm Infants) OR Extremely Preterm Infant) OR Infant, Extremely Preterm) OR Infants, Extremely Preterm) OR Preterm Infant, Extremely) OR Preterm Infants, Extremely) OR Extremely Premature Infants**			

Latest search date: 1 December 2018

Searches:

Search Criteria #1 **AND** Search Criteria #2

Search Criteria #1 **AND** Search Criteria #3

Search Criteria # 2 **AND** Search Criteria #3
